# Geometric De-noising of Protein-Protein Interaction Networks

**DOI:** 10.1371/journal.pcbi.1000454

**Published:** 2009-08-07

**Authors:** Oleksii Kuchaiev, Marija Rašajski, Desmond J. Higham, Nataša Pržulj

**Affiliations:** 1 Department of Computer Science, University of California, Irvine, California, United States of America; 2 Faculty of Electrical Engineering, University of Belgrade, Belgrade, Serbia; 3 Department of Mathematics, University of Strathclyde, Glasgow, United Kingdom; National Center for Biotechnology Information (NCBI), United States of America

## Abstract

Understanding complex networks of protein-protein interactions (PPIs) is one of the foremost challenges of the post-genomic era. Due to the recent advances in experimental bio-technology, including yeast-2-hybrid (Y2H), tandem affinity purification (TAP) and other high-throughput methods for protein-protein interaction (PPI) detection, huge amounts of PPI network data are becoming available. Of major concern, however, are the levels of noise and incompleteness. For example, for Y2H screens, it is thought that the false positive rate could be as high as 64%, and the false negative rate may range from 43% to 71%. TAP experiments are believed to have comparable levels of noise.

We present a novel technique to assess the confidence levels of interactions in PPI networks obtained from experimental studies. We use it for predicting new interactions and thus for guiding future biological experiments. This technique is the first to utilize currently the best fitting network model for PPI networks, geometric graphs. Our approach achieves specificity of 85% and sensitivity of 90%. We use it to assign confidence scores to physical protein-protein interactions in the human PPI network downloaded from BioGRID. Using our approach, we predict 251 interactions in the human PPI network, a statistically significant fraction of which correspond to protein pairs sharing common GO terms. Moreover, we validate a statistically significant portion of our predicted interactions in the HPRD database and the newer release of BioGRID. The data and Matlab code implementing the methods are freely available from the web site: http://www.kuchaev.com/Denoising.

## Author Summary

Proteins are responsible for much of the biological ‘heavy lifting’ that keeps our cells functioning. However, proteins don't usually work alone; instead they typically bind together to form geometrically and chemically complex structures that are tailored for a specific task. Experimental techniques allow us to detect whether two types of proteins are capable of binding together, or ‘interacting’. This creates a network where two proteins are connected if they have been seen to interact, just as we could regard two people as being connected if they are linked on Facebook. Such protein-protein interaction networks have been developed for several organisms, using a range of methods, all of which are subject to experimental errors. These network data reveal a fascinating and intricate pattern of connections. In particular, it is known that proteins can be arranged into a low-dimensional space, such as a three-dimensional cube, so that interacting proteins are close together. Our work shows that this structure can be exploited to assign confidence levels to recorded protein-protein interactions and predict new interactions that were overlooked experimentally. In tests, we predicted 251 new human protein-protein interactions, and through literature curation we independently validated a statistically significant number of them.

## Introduction

### Protein-Protein Interaction Networks

Networks (also called graphs) are used to model natural phenomena studied in computational and systems biology. Nodes in networks represent biomolecules such as genes or proteins, and edges between the nodes indicate interactions between the corresponding biomolecules. These interactions could be of many different types, including functional, genetic, and physical interactions. Understanding these complex networks is a fundamental issue in systems biology. Of particular importance are protein-protein interaction (PPI) networks. In PPI networks, nodes correspond to proteins and two nodes are linked by an edge if the corresponding proteins can interact. The topology of PPI networks can give new insight into the function of individual proteins, protein complexes and cellular machinery as a complex system [1],[2].

Advances in high-throughput techniques such as yeast-2-hybrid (Y2H), tandem affinity purification (TAP), and mass spectrometric protein complex identification (HMS-PCI) are producing a growing amount of experimental PPI data for many organisms [3]–[11]. However, the data produced by these techniques have very high levels of false positives and false negatives. Y2H screens have false negative rates in the range from 43% to 71% and TAP has false negative rates of 15%–50% [12]. False positive rates for Y2H could be as high as 64% and for TAP experiments they could be as high as 77% [12]. Thus, reducing the level of noise in PPI networks and assessing the confidence of each interaction is an essential task.

Two recent studies provided two high quality PPI data sets for Saccharomyces cerevisiae [5],[10]. Gavin et al. [5] defined “socio-affinity” scores measuring the log-odds of the number of times two proteins are observed together, relative to their frequency in the data set. They use not only direct bait-prey connections but also indirect prey-prey relationships. In this, two proteins are each identified as preys in a purification in which a third protein is used as bait. Krogan et al. [10] used machine learning methods, including Bayesian networks and boosted stump decision trees, to define confidence scores for potential interactions. These scores are based on direct bait-prey observations. They used a Markov clustering algorithm to define protein complexes.

Data sets produced by these two groups are very different and thought to contain many false positives. In [11] these two data sets were merged into one set of experimentally based PPIs by analyzing the primary affinity purification data using the purification enrichment (PE) scoring system. Using the set of manually curated PPIs, they showed that this new data set is more accurate than the original individual sets and is comparable to PPIs defined using small scale experimental methods. From the original 12,122 interactions from these two studies in the General Repository of Interaction Data (BioGRID) [13] they discarded 7,504 as being of low confidence. Applying their metric they discovered 4456 new interactions, that were not among the original 12,122 interactions, and produced a set of 9,074 interactions with accuracy comparable to the accuracy of the small scale experiments. In this paper we use this high confidence data set to test our approach.

In recent years several random graph models have been proposed to model PPI networks: Erdös-Rényi random graphs with the same degree distribution as in data [14], scale-free graphs [15], geometric random graphs [16]–[18], and stickiness-index-based models [19]. The technique presented in this paper is one of the first to use a network model of PPI networks for purposes other than just generating synthetic data. We demonstrate that a geometric graph model can be used for assessing the confidence levels of known interactions in PPI networks and predicting novel ones. We apply our technique to de-noise PPI data sets by detecting false positives and false negative interactions. This new approach is compared with existing PPI network post-processing techniques in the final section.

### Geometric Graph Model

Proteins form interactions with each other based on their biochemical properties. Mathematically, we can consider these properties to be dimensions of some abstract metric space. Therefore, PPI networks reside in some biochemical space with finite number of dimensions. Currently, it is hard even to hypothesize about the nature or dimensionality of that space, however in previous work [16]–[18],[20], using various mathematical and computational techniques, we have shown that PPI networks are well modeled by low dimensional *geometric random graphs* [21]. In a geometric random graph, nodes correspond to points distributed uniformly at random in a metric space and edges exist between nodes that are within a chosen distance 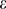 according to a chosen distance norm. Thus, geometric random graphs are a versatile graph family, since they can be constructed using different metric spaces, distance norms, and distance parameter. Many of their properties can be proved theoretically [21]. We choose low-dimensional Euclidean boxes and the Euclidean distance norm to construct geometric random graphs with the number of nodes equal to that of a PPI network; we chose 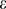 that makes the number of edges in the geometric graph equal to the number of edges in the PPI network. Euclidean space is chosen only as a proof of concept; it is likely that customized models would provide better fits, at the expense of model complexity.

It is well known that geometric random graphs constructed using 2-dimensional Euclidean space cannot contain certain types of induced bipartite subgraphs that appear to be abundant in the currently available PPI networks [21],[22]. However, increasing the dimension of the Euclidean space makes more subgraphs possible, in particular 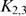, the complete bipartite graph based on two sets of two and three nodes is allowed in three dimensions. Note that there is a bias coming from experimental “spoke” model used for detecting protein interactions [23] which will necessarily introduce small bipartite graphs containing false positives in the data. Also, nothing prevents geometric graphs from being scale-free [24].

The random geometric graph model matches PPI networks in terms of various global and local network properties such as pathlengths, clustering coefficients, relative graphlet frequency distance [16], and graphlet degree distribution [17]. We have also designed an algorithm to test directly whether PPI networks are geometric by embedding them into a low dimensional Euclidean space [18]. The algorithm is based on Multi-Dimensional Scaling [25], with pathlengths playing the role of Euclidean distances. The embedding is “successful” if it assigns to nodes of a network a set of points in space such that adjacent nodes in the network correspond to points that are close in space, whereas non-adjacent nodes correspond to points that are further away in space. Given such an embedding, we are able to reconstruct the original network by choosing a distance cutoff, which also controls sensitivity and specificity [18]. Success may be quantified through Receiver Operator Characteristic (ROC) curve and precision versus recall analysis.

We applied this algorithm on 19 PPI networks of various organisms that were produced by a range of biological techniques with various confidence levels. The algorithm successfully embedded these networks into a low-dimensional space thus supporting the hypothesis that PPI networks are geometric [18].

## Methods

### Overview

A graph 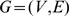, where 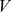 is a set of nodes and 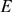 is a set of edges, is called *connected* if for all pairs of nodes 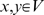 there is a path between them comprised of edges from 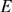. Real PPI networks are not connected, but they usually have one large connected component, which includes most (about 90%) of the network's nodes and edges. For example, the human PPI network obtained from BioGRID (version 2.0.35) [13] has 7,930 proteins with 7,513 of them belonging to the largest connected component. In this paper, we use only the largest connected component, since embedding disconnected components of a graph into space may result in meaningless spatial overlap. Intuitively, it is difficult to see how any algorithm that uses PPI data alone could infer links between members of disconnected components. Hence, in particular, we are not aiming to predict new interactions between members of disconnected components.

We embed the largest connected component of a PPI network into low dimensional space, and compute spatial distances between the embedded nodes. Some nodes are very close in the projection space compared to the average distance between pairs of nodes that are recorded as interacting (true positives obtained from the high-confidence data set). Also, some nodes are far apart compared to the average distance between pairs of nodes that are known, with a certain confidence, not to interact (true negatives). Pairs of nodes that are unusually close to each other, but are not connected in the PPI network, are good candidates for false negatives. On the other hand, pairs of nodes that are connected in the PPI network, but are unusually far apart in the embedding space, are strong candidates for false positives. These are the principles on which we develop our algorithm.

### The Embedding Algorithm

We briefly describe our embedding algorithm. It is based on Multi-Dimensional Scaling (MDS) [25]. Note that MDS is a spectral method, based on eigenvalues and eigenvectors, and in this sense it is similar to algorithms that use the Fiedler vector from the graph Laplacian [26]. However, there is a key difference in the way in which pairwise weights between nodes are interpreted. MDS regards a *larger* pairwise weight between nodes as an indication of more *dissimilarity*.

Given *pairwise Euclidean distances* 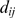 between all pairs of 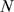 elements in a set, the task is to find locations in *m*-dimensional Euclidean space (vectors 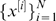 in 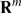) for these elements so that pairwise distances are preserved, i.e., 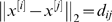 for all 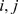. This is not possible, in general, for a given dimension 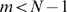, and therefore we want to find the best approximation. If the distance information data respects the triangle inequality, *double centering* gives the symmetric, positive semi-definite matrix 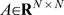,
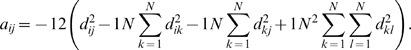
(1)It may be shown that
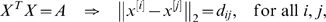
(2)where 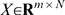 is the matrix whose 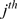 column is 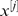. The matrix 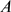 has the real Schur decomposition [27] 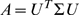, where 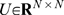 is orthogonal and 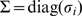. Rows of 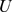 are the eigenvectors of 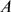 and diagonal entries in 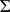 are the eigenvalues of 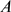 ordered high-to-low. The solution 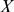 in equation (2) may be computed as 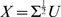.

An embedding into 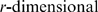 space is found by truncating to the largest 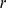 eigenvalues, giving
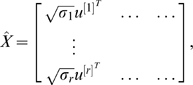
(3)where 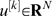 is the 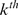 row of 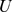. This is the optimal embedding into 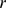 dimensions in the sense that 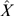 is the closest matrix of rank at most 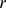 to the exact solution 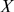, in any orthogonally invariant norm [27].

In PPI networks, we only have {0,1} *connectivity* information, rather than Euclidean distances. This is why we use a function of the pathlength (the length of the shortest path between nodes in the network) in lieu of the Euclidean distance. Our experiments suggest that square root of the graph pathlength is a good function for this purpose. Thus, we use 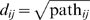, where 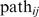 denotes the pathlength between nodes 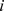 and 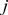. We also set an upper threshold on 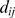. This allows sparsity to be exploited for computational efficiency. Subspace iteration [27] is used to compute eigenpairs of the matrix 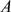 in equation (1). The algorithm typically requires only a few sparse matrix multiplications and the overall complexity is less than the 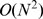 cost of computing pairwise distances between nodes in the new embedding, where 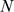 is the number of nodes. For practical details about the algorithm, see [18].

### Geometric De-noising of PPI Networks

Our de-noising approach exploits the fact that high quality PPI networks are well modeled by geometric graphs [16]–[18]. The basic version of our de-noising procedure consists of the following steps:

#### Algorithm 1

- Embed a PPI network into Euclidean space of dimension 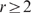.
- Choose a threshold 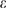.
- Find all “non-edges” (pairs of nodes corresponding to proteins that are not interacting in the PPI network) with Euclidean distance between their embedding 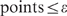. These are our new predicted PPIs (edges).

This procedure may be iterated in the sense that we can add our predictions to the network and re-embed to produce new predictions. In all our experiments for any dimension, this process converged after very few iterations. We used this procedure to test our approach (see section “Testing of geometric de-noising”).

**Figure 1:**
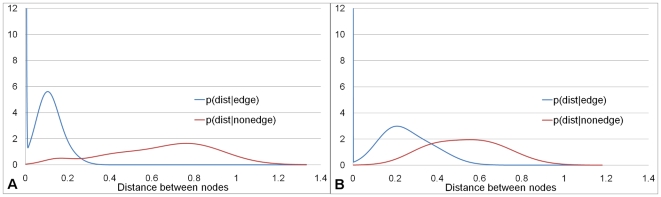
Probability density functions 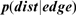 and 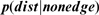. Probability density functions 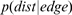 and 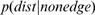 learned from embedding the largest connected components of the following PPI networks into 5-dimensional Euclidean space: (A) the yeast *S. cerevisiae* high confidence PPI network [11] (“Yhigh”); (B) the human PPI network from BioGRID (version 2.0.35) [13] (“HumanBG”). The 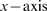 represents the values of the Euclidean distances between pairs of nodes in the embedding; the 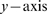 represents the values of probability density functions 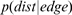 and 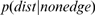.

For real applications, we use a slightly modified procedure in which rather than strictly classifying pairs of nodes into edges (interaction) and non-edges, we assign confidence scores to them reflecting the likelihood for the pairs of nodes to interact. In this manner, we learn the following two probability density functions from the data: 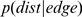 and 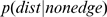, where 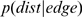 is the probability density function which describes the distribution of distances between pairs of proteins which are known to interact (i.e., form edges in the PPI networks) and 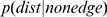 is the probability density function which describes the distribution of distances between pairs of proteins which are not interacting (non-edges in the PPI network). We learn 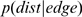 and 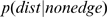 from the data given by the embedding step (see Figure 1 A and B). These densities are modeled as mixtures of three Gaussians and all parameters are learned from the data using the Expectation Maximization algorithm [28]:
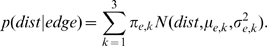
(4)


The density of the distribution 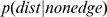 is computed using formula (5) below over all pairs of proteins for which interaction is not known to exist. Note that since the fraction of the real interaction is orders of magnitude lower than the possible number of protein pairs in the network [29], unknown interactions will not have significant effect on this density
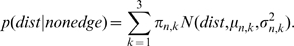
(5)


These are the linear combinations of three Gaussian distributions with means 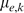 and variances 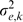 for edges and 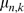 and 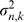 for non edges. The number of mixtures in models (4) and (5) was selected to be 3, since we observed that the histograms corresponding to the densities 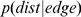 and 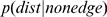 had no more than 3 modes in all of our experiments.

Note that both distributions presented in Figure 1 A and B are bi-modal. Therefore, posteriors 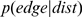 and 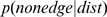 will also be bi-modal (see Figure S1 and Figure S2). This low modality comes comes from the fact that these PPI networks are well modeled even by 2-dimensional geometric random graphs. Intuitively, the smaller the distance between two proteins, the higher the likelihood for them to interact. This is reflected by confidence scores (formula 6), which take into account 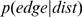 and 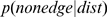 simultaneously and monotonically increase when distance between two proteins decreases (Figure S3).

Our modified procedure may be summarized as follows:

#### Algorithm 2

1 Embed PPI network into Euclidean space of dimension 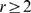.
2 Learn probabilistic densities 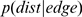 and 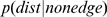 from coordinates of node embedding points in the space.
3 Choose some threshold 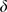.
4 For each pair of nodes with 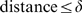 compute its confidence score (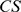).

The confidence score for the pair of nodes 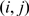 is computed as
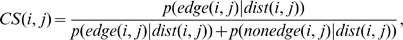
(6)where 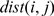 is the distance between points corresponding to nodes 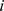 and 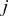 in the embedding and 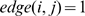 if 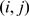 is an edge in the PPI network and 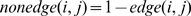. This score is proportional to the likelihood of a pair of nodes to form an edge if all noise that prevents the current PPI network from being a geometric graph is removed.

Using Bayes' rule we compute posterior densities 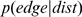 and 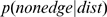:
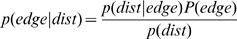
(7)

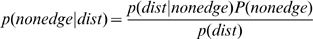
(8)where 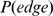 is a prior belief about what fraction of pairs of nodes in the PPI network are true interactions (edges). One can choose different priors to reflect existing knowledge about the density of a particular PPI network. We compute 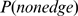 as 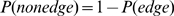. The fraction of real edges among all possible node pairs in real PPI networks is very small. For example, it is estimated that among about 6,000 proteins in the yeast *S.cerevisiae*, there are only 30,000–75,000 interactions [29]–[31], which is a small portion of the maximum possible total of ≈17×10^6^. The human PPI network is estimated to have 154,000–369,000 interactions among 20,000–25,000 proteins [29]. Thus, in reality 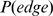 is very small, which helps us avoid many false positives in the network. We do not need to know 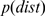, since it can be treated as a normalization constant.

The parameter 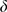 prevents us from assigning confidence scores (CS) to the pairs of nodes that are very far apart and thus are very unlikely to interact. Algorithm 2 could be reduced to Algorithm 1 by choosing an appropriate confidence score threshold value.

### Data

We use two different datasets, one to test our approach and the other to provide a practical application of our method. Since the yeast PPI network described by Collins *et al.* [11] is believed to be of high confidence, we use it to test our approach. The high confidence part of this network consists of 9,074 interactions amongst 1,622 proteins and it is not connected. We take its largest connected component (henceforth denoted by “Yhigh”) which has 8,323 interactions between 1,004 proteins. We use low confidence edges of this network to verify our predictions, i.e., we try to “predict” these low confidence interactions. That is, by true positive, we mean an edge that is predicted by our method and present in the full network described by Collins *et al.* [11]. Analogously, a true negative is a pair of nodes predicted by our method not to interact that does not correspond to any edge in the Collins et al. network [11].

For application purposes, we use the human PPI network downloaded from BioGRID (version 2.0.35), which consists of 23,543 interactions amongst 7,930 proteins. In our analysis, we considered only physical interactions from BioGRID detected by one (or several) of the experimental methods presented in Table S6. We consider only the largest connected component of this network, which contains 23,372 interactions amongst 7,513 proteins (henceforth denoted by “HumanBG”).

## Results

### Testing of Geometric De-noising

We use the PPI network described by Collins et al. [11] to test our approach. This data set is described in the “Data” subsection of “Methods”.

In Figure 1A, we present probability density functions 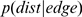 and 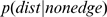 learned from the data given by embedding of “Yhigh” into 5 dimensional Euclidean space. This figure shows that a huge fraction of edges correspond to very close pairs of points in space (a peak very close 0) and most of the non-edges correspond to pairs of nodes with distances about 0.7 between them. This difference between the functions 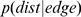 and 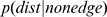 justifies the procedures described in the Methods section to classify pairs of nodes into edges and non-edges based on the distances between them in the embedding.

Our experiments suggest that the choice of dimension is not crucial here. The crucial fact we exploit is that PPI networks are well modeled by low dimensional geometric graphs and the actual value of dimensionality (e.g. 3 or 10) does not change the results much.

**Figure 2:**
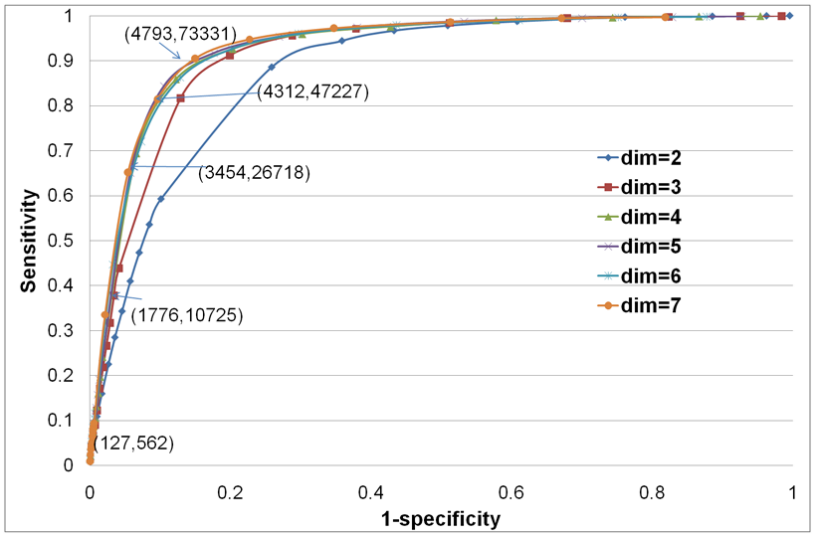
ROC curves for “Yhigh” PPI network for embedding space dimensions of 2 to 7. ROC curves measuring the accuracy of de-noising procedure when applied to “Yhigh” PPI network using embedding space dimensions of 2 to 7. 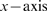 is 1–specificity and 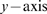 is sensitivity. Numbers in brackets correspond to the numbers of true positives and false positives for a given distance cutoff (TP,FP).

To validate our basic approach, we first test the 2-class classifier performance of Algorithm 1 (see Methods section) using a standard ROC curve analysis. These ROC curves, which are presented in Figure 2 for different embedding space dimensions, were constructed by varying 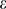 from 0 to the maximum distance between the points in the corresponding embedding space. ROC curves depict relative trade-offs between benefits and costs. For each 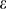, we compute TP (true positives), FP (false positives), TN (true negatives), FN (false negatives), where TP denotes the intersection between the predicted and the low confidence edges, FP denotes the predicted edges which are not in the set of low confidence edges, TN denotes the edges that are neither in the set of predicted edges nor in the set of low confidence edges, and FN stands for the edges which are not predicted, but are present in the set of low confidence edges. For the graph of the ROC curve, the horizontal axis is defined as 1 - *specificity* (or false positive rate), that is, 1−TN/(TN+FP), and the vertical axis is defined as *sensitivity* (true positive rate), TP/(TP+FN).

**Figure 3:**
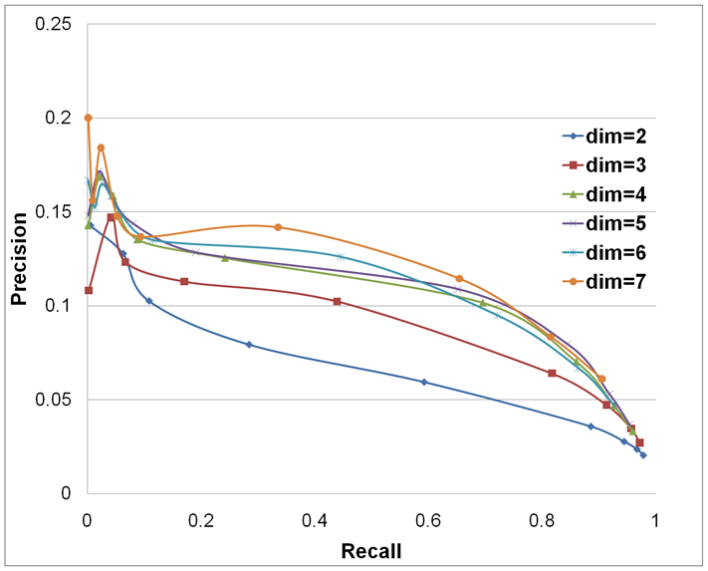
Precision *versus* Recall curves. Precision *versus* Recall curves for “Yhigh” PPI network for embedding space dimensions of 2 to 7. 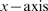 is recall and 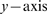 is precision.

Furthermore, in Figure 3 we present precision versus recall analysis, where precision = TP/(TP+FP) and recall = TP/(TP+FN). Note that since we test for presence of interaction amongst all possible pairs of proteins in the largest connected component, the fraction of true positives (interactions) is orders of magnitude lower than the fraction of true negatives (non-interactions) [29]. Therefore, if we predicted interactions completely at random, we should expect less than 1 in 1000 of interaction predictions to be correct, whereas the interaction prediction value (precision) of our method can be about 0.15 at a recall of about 0.35 (see Figure 3). Assuming the estimates of the human PPI network having 154,000–369,000 interactions among 20,000–25,000 proteins [29] is correct, the recall of 0.35 would give us at least 53,900 true interactions (compared to currently available 23,543 human PPIs in BioGRID); in other words, our method has the potential of predicting at least twice as many interactions as there are currently available in BioGRID (at a precision of about 15%).

**Figure 4:**
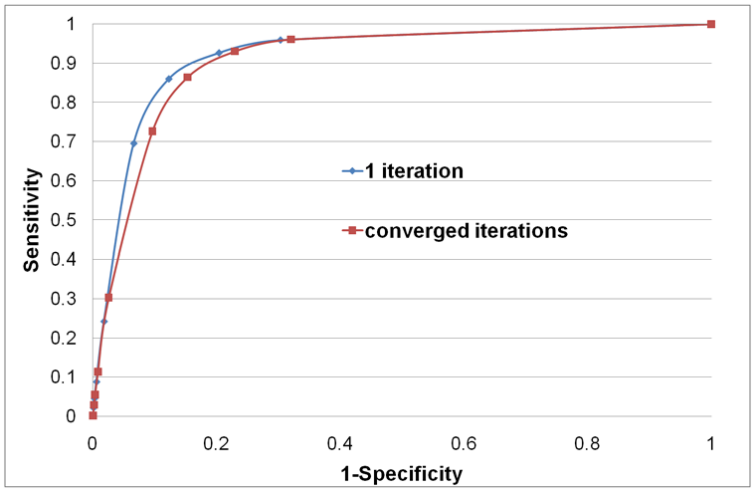
ROC curves for “Yhigh” PPI network with and without iterating embedding and de-noising procedures. The first (blue) ROC curve shows the performance of the de-noising procedure applied to “Yhigh” PPI network using embedding space dimension of 4. The second (red) ROC curve shows the performance after iterating the embedding and de-noising procedures until convergence. 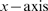 is 1–specificity and 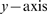 is sensitivity.

For a given value of 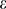, nothing prevents us from adding our predictions to the PPI network we started from and repeating our procedure. We have observed that this iterative procedure always converges. For small values of 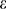, it requires only few iterations (about 10, depending on the network and the space dimension used) to converge. In Figure 4, we present two ROC curves for the cases where we stopped the procedure after the first iteration and for the case where for each 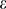, we iterated until convergence (embedding into space of dimension 4 is presented). As can be seen from this figure, the ROC curve for the iterative procedure is only slightly worse than when we stopped the procedure after the first iteration. Therefore, the approach is stable not only in the sense that it converges in few iterations, but also in the sense that the accuracy loss is insignificant during iterations.

**Figure 5:**
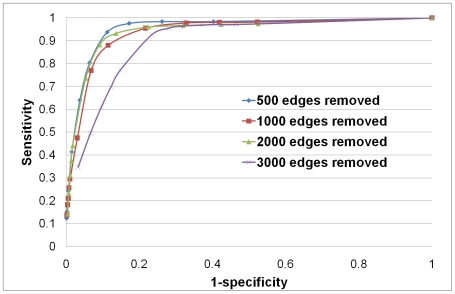
ROC curves for recovering deleted edges. ROC curves for the experiments in which 500, 1000, 2000 and 3000 edges from “Yhigh” network were removed at random and then recovered using the de-noising procedure. 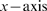 is 1–specificity and 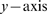 is sensitivity.

To further demonstrate the performance of our approach we perform another experiment that models the incompleteness of current PPI data sets. We take the “Yhigh” network and remove 500, 1000, 2000 and 3000 edges and try to recover these edges using our procedure. The results, presented as ROC curves, are shown in the Figure 5.

These results are encouraging. For example, for dimension 7 of the embedding space (see Figure 2), the area under the ROC curve is 0.9 and we can achieve specificity of 85% and sensitivity of 90%. This corresponds to the false positive rate 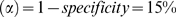 and false negative rate 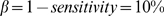. Since we are predicting low-confidence interactions from [11], our true FP and FN rates could be a little higher that measured in this experiments. However, TAP and Y2H false positive and negative rates are believed to be at about 64% and 50% correspondingly [12]. In the absence of further information, it is reasonable to assume that these rates are approximately the same on all parts of the network, including its largest connected component. Hence, for the largest connected component of the network our method has significantly better FP and FN rates than these two experimental techniques.

### Application to Human PPI Network

We apply our method to predict novel interactions in the human PPI network “HumanBG” (see the “Data” subsection of “Methods”).

Using Algorithm 2 presented in “Methods” section, we compute confidence scores for all possible pairs of proteins with Euclidean distance between the corresponding points in the embedding being lower than 0.4. Figure 1B shows 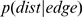 and 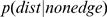 in the case of embedding into the 5-dimensional space. Since the overlap between these two densities is small and most of the interacting protein pairs have distances between their corresponding points very close to 0, we can assign confidence scores to the interactions (existing and potential) in this PPI network. The value of 0.4 of 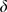 was chosen because, as illustrated in Figure 1B, most node pairs with embedding points at distance 0.4 or higher are non-edges. For other PPI datasets, a realistic value for 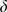 may be different.

There are 2,838 edges (about 12% of all edges in the network) that correspond to protein pairs with endpoints further away than 0.4 in the embedding. We refer to these edges as our candidates for false positive PPIs. In the “HumanBG” network, about 72% of interactions correspond protein pairs that share at least one “cellular localization” Gene Ontology (GO) term [32]. Proteins with different cellular localizations are believed to be less likely to interact. We confirm this by verifying that for our false positive interaction candidates, this rate is about 66%, which is less than that of the entire PPI network. Hence, we suggest that the interactions predicted by our method not to interact that do not share “cellular localization” GO terms are strong candidates for false positives (Table S7).

Next, we examine all possible pairs of nodes that were assigned confidence scores (CS) of 0.975 or higher. There are 1,685 such pairs. Not surprisingly, most of them (1,434) are edges in the “HumanBG” network. We refer to these edges as high confidence edges. The remaining 251 pairs of nodes with 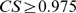 do not correspond to edges in the “HumanBG” network and therefore, we consider them as our high confidence predictions (presented in Table S1). The human PPI network from BioGRID is one of the most complete PPI datasets for human. However, to validate some of our predictions, we also examined human PPI interactions from Human Protein Reference Database (HPRD) [33]. We validated 12 of our predictions (that we predicted using BioGRID) by finding them in HPRD. Given a huge amount of possible protein pairs in the human PPI network (about 28 million) such overlap between our predictions and HPRD is extremely unlikely to have happened at random: our validation of 12 interactions is highly statistically significant with the p-value of 7×10^−8^ (see Text S1 for details). When this paper was almost finished, a new release of BioGRID (version 2.0.50) was made available for download and 5 of our predictions appeared in it; 4 of these 5 interactions were present before in HPRD and 1 was a new interaction. Therefore, in total, 13 of our predictions are validated by HPRD or the newest version of BioGRID (version 2.0.50) or by both of these databases (presented in Table S2). Furthermore, our method predicts that proteins POP5 and POP1 interact, which is supported by the HPRD database; moreover, Krogan *et al.* [10] detected a physical interaction between proteins POP5 and POP1 in yeast. Also, we predict that proteins CAR1 and MDH1 interact in human and these two proteins were found to interact in yeast using Affinity Capture-MS method [34].

Similar to the study by Yu and Finley [35], we investigate the biological significance of our PPI predictions using regular (not slim) GO terms and KEGG pathways; in addition, we use a literature search and text mining tool. First, we examine how many predicted interaction pairs share common Gene Ontology (GO) terms [32]. Since proteins that are involved in the same biological process and/or share the same cellular localization are more likely to interact, this statistic can give us a better idea of the quality of our predictions. Initially, we take into account only those protein pairs in which both proteins are annotated with at least one GO term, ignoring “root” GO terms (GO:0008150 for biological process and GO:0005575 for cellular component). Among our 251 predictions, 92 protein pairs had at least 1 unannotated protein, thus we had complete GO data only for 159 protein pairs. Out of these protein 159 pairs, 105 (66%) have at least 1 common GO term that corresponds to “biological process,” or “cellular localization” (presented in Table S3). The statistical significance, measured as a p-value, of this result is 7.26*10^−8^ (see Text S1 for details).

GO terms that correspond to “cellular localization” could be very general; many proteins may share the same “cellular localization,” without interacting. Thus, to further investigate the biological significance of our predictions we disregard from our analysis GO terms related to “cellular localization” and consider only known GO terms related to “biological process.” Out of our 251 high confidence predictions, this restriction results in 129 protein pairs having both interactors in the GO “biological process” category. Out of these 129 pairs, 55 pairs have at least one such GO term in common (presented in Table S4). The statistical significance of this result (p-value) is 1.4*10^−8^ (see Text S1 for details).

To further investigate the biological significance of our predictions, we count how many of our 251 predictions consist of proteins involved in the same KEGG pathway [36]. As of March 2009, there were 205 pathways for human in the KEGG database. The number of genes involved in the same pathway varies greatly from 1 to 467, with the average number of genes in the same pathway being 67 genes. Yu and Finley [35] found that for their high confidence scored dataset of human protein interactions (that they termed “HCS”), about 10% of the interactions belong to the same KEGG pathway. We found that out of our 251 high confidence predictions, 26 (i.e., about 10%) correspond to pairs of proteins where both proteins participate in some of the KEGG pathways. Out of these 26 predicted interactions, 12 (i.e., about 46%) correspond to protein pairs participating in the same pathway (Table S5). Note however, that pathways have a “linear” structure in a PPI network, i.e., they are “stretched” along long paths of proteins between receptors and transcription factors. Thus, the “end-nodes” of pathways (i.e., receptors at one end and transcription factors at the other) can be far away in a PPI network [37]. Since our method for predicting PPIs is based on the PPI network's spatial embedding that relies on the proximity of proteins along shortest paths in a PPI network, the “linearity” of pathways in PPI networks implies that our method is not geared towards predicting interactions belonging to the same pathway. Nevertheless, our success rate for predicting such interactions is about 5%, which is particularly encouraging given the fact that only about 10% of all PPIs in a PPI network belong to the same pathway [35].

Finally, we use literature search and text mining service CiteXplorer [38] to find out how often protein pairs that correspond to our high confidence predictions are mentioned in the abstract of the same paper in PubMed. For 32 of our 251 predictions, CiteXplorer found at least one article mentioning both proteins simultaneously.

## Discussion

High levels of inherent noise in experimental techniques for detecting protein-protein interactions has stimulated the development of computational techniques for assessing their confidence levels and prediction of new interactions. In the realm of interaction prediction, some approaches use only primary structure of proteins, or protein domains [39]–[43]. Others exploit features such as messenger RNA co-expression, co-essentiality, and co-localization of proteins [44]. There exist approaches that use protein structure, functional annotation, co-localization information, etc. [45]. These computational techniques usually have better accuracy than high-throughput experiments. For example, PIPE [40] has sensitivity of 61% for detecting any yeast protein-protein interaction with 89% specificity. However, computational requirements for this algorithm do not allow for large-scale computational experiments (evaluating the reliability of every possible link). Other approaches, such as PreSPI [39], also have good specificity of 73.20% and sensitivity of 96.77%. Table 1 presents commonly used methods for predicting protein interactions [39]–[43]. Note that most of them are sequence-based, or utilize information such as functional annotation. As Table 1 shows, our method has higher sensitivity than methods which utilize only sequences [39],[40],[43]. When additional information (such as functional annotation, biochemical properties of proteins, etc.) is available other methods might outperform our approach. However, this additional information is available only for a limited set of proteins which significantly limits application of these methods. It is important to note that our method does not need any particular knowladge about individual proteins (even sequences) and therefore is a novel and independent source of information about PPI interactions.

**Table 1:** Computational methods for predicting protein-protein interactions.

Method	Sensitivity	Specificity	Input	Comments
PreSPI [39]	77%	95%	Learning set of protein sequence pairs known to be interacting or non-interacting. Protein sequences for interaction prediction.	Requires a learning set with interacting and non-interacting protein pairs containing different domains. Once the classifier is trained, then it requires as input only protein sequences of protein pairs for which interaction is being predicted. Applied to yeast.
Ma et al. [41]	91%	86%	Training (i.e., learning) set of protein sequence pairs known to be interacting or non-interacting. Protein sequences for interaction prediction.	Requires a training set with interacting and non-interacting protein pairs. Requires Matlab seqtool for getting protein biochemical properties. Once the classifier is trained, then it requires as input only protein sequences of protein pairs for which interaction is being predicted. Applied to yeast.
Lee et al. [42]	94%	97%	For both proteins that we are checking for interaction: 1) Functional category; 2) Co-localization; 3) Topology within PPI network.	Application is limited only to protein pairs with known functional and localization annotations. Applied to yeast.
PIPE [40]	61%	89%	Protein sequences.	Reported to be weak for detecting novel interactions among genome wide large-scale data sets [40]. Applied to yeast.
Chen and Liu [43]	78%, 77%, 79%	37%, 65%, 62%	Training (i.e., learning) set of protein sequence pairs known to be interacting or non-interacting. Protein sequences for interaction prediction.	Requires a training set with interacting and non-interacting protein pairs. It is a protein domain-based approach. It uses one of the following three types of classifiers: a) Decision tree, b) Neural network c) MLE. This is why three values are reported for sensitivity and specificity, respectively. Applied to yeast.
**Our Method**	90%	85%	Protein-protein interaction network.	Based solely on PPI network topology. Does not require any knowledge about particular proteins. Is it generally applicable to any organism.

The field “Method” refers to a particular method either by the method name or by the last names of its authors. Fields “Sensitivity” and “Specificity” contain values as reported by the authors of particular methods. “Input” field describes what kind of input is expected by the algorithm and “Comments” field contains general comments about usage of the algorithm.

There exist techniques that can be utilized to remove false positives from the existing data without predicting novel interactions [23],[35]. Sometimes such approaches are based on logistic regression and require several PPI data sets originating from different experiments; they are able to detect parts of PPI networks of the highest quality by using overlaps of the data sets. Although these techniques can be used to propose high quality PPIs, the completeness of the data still remains an issue and can be resolved only by combining multiple experimental datasets, or by additional wet-lab experiments. Since there does not exist a gold standard PPI network for any organism, it is hard to judge which of the interactions from those reported by these methods to be of low-confidence are true interactions and which are false-positives. The same, is true for our method. Hence, we believe that all computationally predicted false positives should be re-tested experimentally.

Similar to our method, there exists a technique for predicting novel PPIs based on the topology of a PPI network [46]. However, that approach is based on a “maximal clique” that potentially can lead to a higher rate of false positives than that of the “spoke model” [23]. Finally, Chen *et al.* [47] devised a topology-based algorithm called IRAP to detect false positives and false negatives in yeast, fly and worm. In their work Chen *et al.* [47] focused only on Y2H-derived experimental datasets, whereas the “HumanBG” network in the focus of our study contains PPIs derived from all possible techniques (available in BioGRID) used to detect physical interactions (see Table S7). Also, unlike IRAP our method actually evaluates the reliability of every possible link. For a review of the methods used for PPI networks de-noising see [48].

Our method uses only PPI network topology for detecting both false positives and false negatives (predicting novel interactions). Unlike most of the methods for detecting false positives, our algorithm does not require several PPI datasets. Also, unlike most methods for predicting novel interactions, it does not need any a priori information about individual proteins, such as binding domains, structure, function, chemical properties, or sequence. On our testing set, we can achieve specificity of 85% and sensitivity of 90% (see ROC curves in Figure 2) and our method can be applied to large-scale network experiments. This overall performance is better than that of biological experimental techniques and is comparable to that of Yu and Finley [35]. However, while Yu and Finley only assess confidence of the existing interactions, our method is also capable of predicting novel ones (Table S1).

It is important to note that the coordinates of the nodes that we get from the embedding do not represent proteins' relative locations in 3-dimensional space in the cell in any way. Instead, the dimensions of the target space might correspond to various bio-chemical properties. Our approach does not need information about what the target space's dimensions represent, nor any knowledge of space dimensionality. Finding optimal dimensionality of this space and the bio-chemical meaning for the dimension is an open research question.

## Supporting Information

Text S1 Supplementary Information for: Geometric de-noising of protein-protein interaction networks
(0.05 MB PDF)

Table S1 All 251 high confidence predictions.
(0.21 MB DOC)

Table S2 Protein-protein interaction predictions validated in HPRD, newest version of BioGRID (2.0.50) or in both databases.
(0.03 MB DOC)

Table S3 Protein-protein interaction predictions where both proteins in the pair share at least one GO term corresponding to the “biological process” or “cellular component”.
(0.14 MB DOC)

Table S4 Protein-protein interaction predictions where both proteins in the pair share at least one GO term corresponding to the “biological process”.
(0.07 MB DOC)

Table S5 Protein-protein interaction predictions where both proteins participate in the same KEGG pathway.
(0.04 MB DOC)

Table S6 Predicted false positives.
(1.10 MB DOC)

Table S7 Experimental techniques from BIOGRID capable of detecting physical interactions between proteins.
(0.04 MB DOC)

Figure S1 Probabilistic density p(edge|dist). x axis corresponds to distances between pairs of nodes, y value of the density. Note, that in this plot normalization constant from formula (7) in the main paper is not taken into account.
(0.02 MB TIF)

Figure S2 Probabilistic density p(nonedge|dist). x axis corresponds to distances between pairs of nodes, y value of the density. Note, that in this plot normalization constant from formula (8) in the main paper is not taken into account.
(0.02 MB TIF)

Figure S3 Confidence scores for “HumanBG network”. x axis corresponds to distances between pairs od nodes, y axis corresponds to the assigned confidence scores.
(0.02 MB TIF)
