# Supplementary material for: Geometric De-noising of Protein-Protein Interaction Networks
Source: PLoS Comput Biol. 2009 Aug 7;5(8):e1000454. doi: 10.1371/journal.pcbi.1000454 (PMC2711306; doi:10.1371/journal.pcbi.1000454)
Supplement: Text S1 — Supplementary Information for: Geometric de-noising of protein-protein interaction networks (0.05 MB PDF) [file pcbi.1000454.s001.pdf]

Supplementary Information for: Geometric de-noising of protein-protein interaction networks

Oleksii Kuchaiev[1], Marija Rašajski[1,2],Desmond J. Higham[3], Nataša Pržulj*[1]

1 Department of Computer Science, UC Irvine, Irvine, CA, 92697, USA
2 Faculty of Electrical Engineering, University of Belgrade Belgrade, Serbia
3 Department of Mathematics, University of Strathclyde, Glasgow G1 1XH, UK
***** Corresponding author: natasha@ics.uci.edu

Statistical Significance

As we describe in the "Results" section of the paper, out of 251 our "high confidence" (with $CS >= 0.975$) predictions we found 12 in the HPRD database. To examine the statistical significance of this result we need to answer the following question. Given the "HumanBG" network and the list of interactions from HPRD database, how likely it is to get 12 or more protein pairs which are present in HPRD database if we select 251 pairs of proteins in HumanBG network at random? We use the standard model of sampling without replacement to answer this question. The p-value we are interested in equals to the area under the tail of the hypergeometric distribution:

$$p = 1 - F(x-1|M,K,N) = 1 - \sum_{i=0}^{x-1} \frac{\binom{K}{i}\binom{M-K}{N-i}}{\binom{M}{N}}$$

The parameters we need are: M is the total number of pairs in the HumanBG network, K - number of interactions in the HPRD database, N - number of pairs we sample and x=12 is the number of sampled pairs which are present in the HPRD. In our case, since HumanBG has 7513 nodes, the total number of node pairs is 28218828. The number of interactions in the HPRD database is $K = 34119$. And our p-value is then about 7.5×10^{-8}.

To examine the statistical significance for sharing common GO terms, we again used the standard model of sampling without replacements. Note, that in our analysis we take into account only those protein pairs in which both proteins are annotated with at least one GO term, which is not a "root" GO term (root GO terms are: GO:0008150 for biological process and GO:0005575 for cellular component). The number of nodes in HumanBG network annotated with at least one GO term which correspond to "biological process" or "cellular

component" is 6278 and the total number of pairs is $M = 19703503$. Total number of pairs of nodes in HumanBG network which share at least 1 such GO term in common is $K = 7400424$. Out of our 251 high confidence predictions 92 have at least one protein unannotated and therefore we have complete data only for $N = 251 - 92 = 159$ protein pairs. Out of these 159 protein pairs, 105 share at least 1 GO term in common. The p-value of this result is then $p = 1 - F(104|19703503, 7400424, 159) = 7.26 \times 10^{-8}$. About 63% of these 105 predictions correspond to protein pairs which share more than 1 GO term in common. For details see Table S3.

Since "cellular localization" GO terms can be very general, it is likely that some proteins can share the same cellular localization but do not interact. Therefore, in our next analysis we disregarded GO terms related to "cellular localization" and condidered only terms corresponding to the "biological process". In "HumanBG" network, there are 5864 nodes annotated with such GO terms and the total number of pairs is then $M = 17190316$. Out of these M pairs, $K = 1354495$ share at least one GO term in common. Out of our 251 predictions in this case we have complete data only for $K = 129$ of them. Out of these 129 protein pairs, 55 pairs share at least one GO term which refers to "biological process". The statistical significance of this result is then $p = 1 - F(54|17190316, 1354495, 129) = 1.4 \times 10^{-8}$. About 45% of these 55 predictions correspond to protein pairs which share more than 1 GO term in common. For details see Table S4.
