# Supplementary material for: Geometric De-noising of Protein-Protein Interaction Networks
Source: PLoS Comput Biol. 2009 Aug 7;5(8):e1000454. doi: 10.1371/journal.pcbi.1000454 (PMC2711306; doi:10.1371/journal.pcbi.1000454)
Supplement: Table S1 — All 251 high confidence predictions. (0.21 MB DOC) [file pcbi.1000454.s002.doc]

Table S1: All 251 high confidence predictions.

| **Official Symbol A** | **Official Symbol B** |
| --- | --- |
| SRP14 | DHX57 |
| SRP14 | GNL2 |
| TAG | RRM2 |
| TAG | RRM2B |
| CCL19 | CCL25 |
| CCL20 | XCL1 |
| CCL20 | XCR1 |
| CCR6 | XCL2 |
| PRIMA1 | COLQ |
| SIM1 | SIM2 |
| SLC7A11 | SLC7A10 |
| HSPC142 | CXORF53 |
| CXORF53 | KIAA0157 |
| SLC7A11 | SLC7A5 |
| SLC7A11 | SLC7A7 |
| SLC7A10 | SLC7A8 |
| SLC7A5 | SLC7A8 |
| SLC7A7 | SLC7A8 |
| SLC7A11 | SLC3A1 |
| POU3F2 | POU3F3 |
| FLJ23588 | SRY |
| UTY | UTX |
| TNFRSF18 | TNFRSF4 |
| TNFRSF18 | TNFRSF7 |
| TNFRSF4 | TNFRSF9 |
| TNFSF7 | TNFRSF9 |
| SIVA | TNFRSF9 |
| TNFSF7 | TNFSF18 |
| SIVA | TNFSF18 |
| RRM2 | SCOTIN |
| RRM2B | SCOTIN |
| RRM2 | WWOX |
| RRM2B | WWOX |
| RRM2 | ZNF148 |
| RRM2B | ZNF148 |
| RRM2 | CARM1 |
| RRM2B | CARM1 |
| RRM2 | CABLES1 |
| RRM2B | CABLES1 |
| RRM2 | GNL3 |
| RRM2B | GNL3 |
| RRM2 | EEF2 |
| RRM2B | EEF2 |
| RRM2 | IFI16 |
| RRM2B | IFI16 |
| RRM2 | PLAGL1 |
| RRM2B | PLAGL1 |
| TESK1 | TPD52L1 |
| SPHK1 | MGC11134 |
| SPHK1 | KIAA1838 |
| SPHK1 | FBXO28 |
| SPHK1 | USP53 |
| SPHK1 | ZA20D3 |
| SPHK1 | SOX30 |
| SPHK1 | TNIK |
| CRHBP | CRHR2 |
| TESK1 | TESK2 |
| TESK1 | FOXK1 |
| TESK1 | PDE3B |
| TORC2 | KIF5C |
| AANAT | GP1BB |
| AANAT | HMGN1 |
| GP1BB | HMGN1 |
| GP1BB | INPP5A |
| HMGN1 | INPP5A |
| SMTN | MYL1 |
| SMTN | PCYT1B |
| HSMPP8 | CALB1 |
| FLJ20125 | PCF11 |
| F25965 | EFHC1 |
| PTK9L | CAPZA3 |
| SRP68 | PARG |
| SRP68 | PDE5A |
| 1300007L22RIK | MDH1 |
| ACTG | MDH1 |
| MASTL | MDH1 |
| RGD1309427 | MDH1 |
| CAR1 | MDH1 |
| LOC499110 | MDH1 |
| GLUD1 | MDH1 |
| MDH1 | CACNB1 |
| MDH1 | DLGH4 |
| WISP1 | 39706 |
| CLPX | MLSTD2 |
| FOXP2 | ACTL6B |
| HLA-DPB1 | PIP |
| BAT4 | SLU7 |
| SPRR3 | PSORS1C2 |
| 39706 | CHST12 |
| CDH18 | CDH19 |
| RPP21 | RPP14 |
| POP4 | RPP14 |
| RPP30 | RPP25 |
| RPP38 | RPP25 |
| CRHBP | UCN3 |
| EXOSC2 | PARN |
| SSTR4 | MRGPRX2 |
| ATPAF2 | ATPAF1 |
| GOLGB1 | BET1L |
| CHRNA2 | CHRNA5 |
| CHRNA2 | CHRNA3 |
| TNFRSF7 | PPIL5 |
| NQO3A2 | CYC1 |
| COX4I2 | CYC1 |
| ALS2CL | ZFYVE20 |
| CD84 | SLAMF7 |
| BFSP1 | UPP2 |
| MED18 | SURF5 |
| SURF5 | THRAP2 |
| SURF5 | THRAP6 |
| SURF5 | CRSP8 |
| SURF5 | MED31 |
| MED18 | MGC88387 |
| THRAP2 | MGC88387 |
| THRAP6 | MGC88387 |
| CRSP8 | MGC88387 |
| MED31 | MGC88387 |
| MED18 | MED8 |
| THRAP2 | MED8 |
| THRAP6 | MED8 |
| CRSP8 | MED8 |
| MED31 | MED8 |
| EAF1 | EAF2 |
| CRHBP | CRH |
| ND2 | SH3MD1 |
| SLU7 | ZBTB5 |
| SLC27A4 | MTX1 |
| PACAP | GOLGA3 |
| SLU7 | MGC11257 |
| SLU7 | ZNF337 |
| SLU7 | C21ORF91 |
| SLU7 | FAM13C1 |
| SLU7 | KIAA1539 |
| SLU7 | FLJ13149 |
| SLU7 | FLJ20291 |
| SLU7 | C1ORF111 |
| LOC55565 | MGC39633 |
| SLU7 | DUSP13 |
| SLU7 | TTC14 |
| ACTL6B | FOXP1 |
| DAZ1 | DAZAP1 |
| KRTHA1 | PCDHB5 |
| KLF3 | FKSG14 |
| BAZ2B | FKSG14 |
| TESK1 | PDCL2 |
| SLC7A11 | FAM57A |
| SLC7A8 | FAM57A |
| TP53RK | C20ORF35 |
| TP53RK | PCGF1 |
| TP53RK | CPNE7 |
| PDCD6 | ALG2 |
| RMRP | POP1 |
| POP5 | POP1 |
| MYL1 | MIB2 |
| PCYT1B | MIB2 |
| RPS6KC1 | BAMBI |
| RPS6KC1 | NUDT3 |
| MYL1 | DVL1L1 |
| PCYT1B | DVL1L1 |
| ROM1 | CNGB1 |
| MKL1 | ETV4 |
| RPL18 | AZIN1 |
| HAAO | GAD2 |
| GPR143 | GPSM3 |
| RGS10 | GNRH1 |
| GP5 | GP9 |
| GHRHR | MLNR |
| EML1 | GRID2IP |
| FLJ23588 | GSK3A |
| GUCA2B | GUCA1B |
| NR6A1 | HESX1 |
| HIC2 | HTN1 |
| CINP | IL11 |
| CLUAP1 | IL11RA |
| SLC25A17 | ABCD2 |
| PXMP4 | ABCD2 |
| SPHK1 | ITPK1 |
| SPRR3 | KRT2A |
| ZDHHC23 | ARG2 |
| HMOX1 | ARG2 |
| TMED1 | KIAA0256 |
| TMED1 | RPLP0 |
| TMED1 | MTF2 |
| TMED1 | SAP130 |
| TMED1 | PCDH1 |
| TMED1 | PRDM4 |
| TMED1 | GSC |
| CLUAP1 | C18ORF54 |
| IL11 | C18ORF54 |
| CLUAP1 | C20ORF72 |
| IL11 | C20ORF72 |
| CLUAP1 | ACMSD |
| IL11 | ACMSD |
| CLUAP1 | OTUB2 |
| IL11 | OTUB2 |
| CLUAP1 | COX7A2L |
| IL11 | COX7A2L |
| CLUAP1 | CCDC14 |
| IL11 | CCDC14 |
| CLUAP1 | FLJ20244 |
| IL11 | FLJ20244 |
| CLUAP1 | MGC29875 |
| IL11 | MGC29875 |
| CLUAP1 | PORIMIN |
| IL11 | PORIMIN |
| CLUAP1 | ZSWIM2 |
| IL11 | ZSWIM2 |
| CLUAP1 | AKR1C3 |
| IL11 | AKR1C3 |
| CLUAP1 | NDUFB9 |
| IL11 | NDUFB9 |
| CLUAP1 | MR-1 |
| IL11 | MR-1 |
| CLUAP1 | TMEM29 |
| IL11 | TMEM29 |
| SIM2 | AHRR |
| PAX1 | VIP |
| PAX1 | CGI-51 |
| K5B | TRNA |
| HIST2H4 | HIST1H2AE |
| NR6A1 | JMJD2A |
| NR6A1 | NR1D1 |
| NR6A1 | NR1D2 |
| MAML2 | WDR12 |
| MAML2 | MAML3 |
| MAML2 | DLL4 |
| NPFF | NPY5R |
| PAN3 | PAIP1 |
| PAN3 | PAIP2 |
| MEOX1 | SOX10 |
| ABCD2 | PEX11A |
| ABCD2 | PEX11B |
| ABCD2 | PEX16 |
| SLC7A8 | BAAT |
| ABCD2 | PEX3 |
| PIP5K3 | TM9SF2 |
| SPRY4 | HRAS1 |
| BFSP1 | UPP1 |
| BFSP1 | OSBP2 |
| EFHC1 | ZNF414 |
| EFHC1 | HYI |
| EFHC1 | C10ORF82 |
| EFHC1 | KIAA1510 |
| FLJ23588 | PNRC1 |
| FLJ23588 | ETV5 |
| FLJ23588 | RAI17 |
| PHLDB2 | SGCG |
| MUS81 | TSSK1 |
| MKL1 | NKX2-3 |
| MGC14126 | KIAA1166 |
| TRAPPC3 | BET3 |
