# Supplementary material for: Geometric De-noising of Protein-Protein Interaction Networks
Source: PLoS Comput Biol. 2009 Aug 7;5(8):e1000454. doi: 10.1371/journal.pcbi.1000454 (PMC2711306; doi:10.1371/journal.pcbi.1000454)
Supplement: Table S2 — Protein-protein interaction predictions validated in HPRD, newest version of BioGRID (2.0.50) or in both databases. (0.03 MB DOC) [file pcbi.1000454.s003.doc]

Table S2: Protein-protein interaction predictions validated in HPRD, newest version of BioGRID (2.0.50) or in both databases.

| **Official Symbol A** | **Official Symbol B** |
| --- | --- |
| POP5 | POP1 |
| MEOX1 | SOX10 |
| CRSP8 | MED8 |
| THRAP6 | MED8 |
| MED31 | MED8 |
| GHRHR | MLNR |
| RPP38 | RPP25 |
| DAZ1 | DAZAP1 |
| TNFRSF4 | TNFRSF9 |
| SURF5 | CRSP8 |
| SURF5 | THRAP6 |
| POP4 | RPP14 |
| SLC7A8 | BAAT |
