# Supplementary material for: Geometric De-noising of Protein-Protein Interaction Networks
Source: PLoS Comput Biol. 2009 Aug 7;5(8):e1000454. doi: 10.1371/journal.pcbi.1000454 (PMC2711306; doi:10.1371/journal.pcbi.1000454)
Supplement: Table S3 — Protein-protein interaction predictions where both proteins in the pair share at least one GO term corresponding to the “biological process” or “cellular component”. (0.14 MB DOC) [file pcbi.1000454.s004.doc]

Table S3: Protein-protein interaction predictions where both proteins in the pair share at least one GO term corresponding to the ``biological process’’ or ``cellular component ’’. In this analysis we take into account only those protein pairs in which both proteins are annotated with at least one GO term, which is not a root GO term (GO:0008150 for biological process or GO:0005575 for cellular component).

| **Official Symbol A** | **Official Symbol B** | **Number of shared terms** | **Shared GO terms** |
| --- | --- | --- | --- |
| POU3F2 | POU3F3 | 12 | GO:0021799 GO:0021799 GO:0021799 GO:0021869 GO:0021869 GO:0021869 GO:0045944 GO:0045944 GO:0045944 GO:0005634 GO:0005634 GO:0005634 |
| CHRNA2 | CHRNA5 | 8 | GO:0030054 GO:0007165 GO:0005886 GO:0016021 GO:0045202 GO:0045211 GO:0006811 GO:0005892 |
| CHRNA2 | CHRNA3 | 8 | GO:0030054 GO:0007165 GO:0005886 GO:0016021 GO:0045202 GO:0045211 GO:0006811 GO:0005892 |
| GP5 | GP9 | 7 | GO:0007596 GO:0007155 GO:0031092 GO:0005886 GO:0005886 GO:0005886 GO:0005887 |
| CCL20 | XCL1 | 6 | GO:0005576 GO:0005615 GO:0007165 GO:0007267 GO:0006935 GO:0006955 |
| SIM1 | SIM2 | 6 | GO:0006355 GO:0005634 GO:0030154 GO:0007165 GO:0007275 GO:0007399 |
| SLC7A5 | SLC7A8 | 6 | GO:0005886 GO:0006520 GO:0006810 GO:0005737 GO:0015804 GO:0015807 |
| MAML2 | MAML3 | 6 | GO:0016607 GO:0045944 GO:0007219 GO:0006350 GO:0006355 GO:0005634 |
| CCL19 | CCL25 | 5 | GO:0005576 GO:0005615 GO:0006935 GO:0006954 GO:0006955 |
| SLC7A7 | SLC7A8 | 5 | GO:0005886 GO:0006520 GO:0006810 GO:0005887 GO:0016323 |
| DAZ1 | DAZAP1 | 5 | GO:0030154 GO:0007275 GO:0005737 GO:0005634 GO:0007283 |
| SLC25A17 | ABCD2 | 5 | GO:0016020 GO:0005777 GO:0005778 GO:0005887 GO:0006810 |
| GOLGB1 | BET1L | 4 | GO:0016020 GO:0005794 GO:0000139 GO:0016021 |
| MED18 | MED8 | 4 | GO:0006350 GO:0000119 GO:0005634 GO:0006355 |
| CRHBP | CRH | 4 | GO:0007565 GO:0007611 GO:0007165 GO:0005625 |
| SIM2 | AHRR | 4 | GO:0006355 GO:0007165 GO:0005634 GO:0005634 |
| CCL20 | XCR1 | 3 | GO:0007165 GO:0006935 GO:0006954 |
| PRIMA1 | COLQ | 3 | GO:0030054 GO:0045202 GO:0042135 |
| SLC7A10 | SLC7A8 | 3 | GO:0006810 GO:0005887 GO:0015804 |
| UTY | UTX | 3 | GO:0055114 GO:0005634 GO:0016568 |
| RRM2B | WWOX | 3 | GO:0005634 GO:0055114 GO:0005737 |
| RRM2B | PLAGL1 | 3 | GO:0005634 GO:0006917 GO:0006917 |
| CDH18 | CDH19 | 3 | GO:0005886 GO:0007156 GO:0016021 |
| MED31 | MED8 | 3 | GO:0005634 GO:0000119 GO:0006355 |
| EAF1 | EAF2 | 3 | GO:0016607 GO:0005634 GO:0006355 |
| PDCD6 | ALG2 | 3 | GO:0016020 GO:0005634 GO:0051592 |
| MKL1 | ETV4 | 3 | GO:0045941 GO:0005634 GO:0006355 |
| PXMP4 | ABCD2 | 3 | GO:0016020 GO:0005777 GO:0005778 |
| NR6A1 | JMJD2A | 3 | GO:0006355 GO:0005634 GO:0006350 |
| NR6A1 | NR1D1 | 3 | GO:0006355 GO:0005634 GO:0006350 |
| NR6A1 | NR1D2 | 3 | GO:0006355 GO:0005634 GO:0006350 |
| ABCD2 | PEX11A | 3 | GO:0016020 GO:0005777 GO:0005778 |
| ABCD2 | PEX11B | 3 | GO:0016020 GO:0005777 GO:0005778 |
| ABCD2 | PEX16 | 3 | GO:0016020 GO:0005777 GO:0005778 |
| ABCD2 | PEX3 | 3 | GO:0016020 GO:0005777 GO:0005778 |
| CCR6 | XCL2 | 2 | GO:0007165 GO:0006935 |
| SLC7A11 | SLC7A10 | 2 | GO:0016020 GO:0006810 |
| SLC7A11 | SLC7A5 | 2 | GO:0006810 GO:0016021 |
| SLC7A11 | SLC7A7 | 2 | GO:0006810 GO:0006865 |
| SLC7A11 | SLC3A1 | 2 | GO:0016020 GO:0006810 |
| TNFRSF4 | TNFRSF9 | 2 | GO:0016020 GO:0005887 |
| RRM2 | WWOX | 2 | GO:0055114 GO:0005737 |
| RRM2B | CARM1 | 2 | GO:0005634 GO:0005737 |
| RRM2 | CABLES1 | 2 | GO:0005737 GO:0005829 |
| RRM2B | CABLES1 | 2 | GO:0005634 GO:0005737 |
| RRM2B | IFI16 | 2 | GO:0005634 GO:0005737 |
| TESK1 | TESK2 | 2 | GO:0006468 GO:0007283 |
| SRP68 | PARG | 2 | GO:0005737 GO:0005634 |
| FOXP2 | ACTL6B | 2 | GO:0005634 GO:0005634 |
| RPP21 | RPP14 | 2 | GO:0008033 GO:0005634 |
| POP4 | RPP14 | 2 | GO:0008033 GO:0005634 |
| RPP30 | RPP25 | 2 | GO:0005634 GO:0008033 |
| RPP38 | RPP25 | 2 | GO:0005634 GO:0008033 |
| EXOSC2 | PARN | 2 | GO:0005737 GO:0005634 |
| SSTR4 | MRGPRX2 | 2 | GO:0007165 GO:0005886 |
| COX4I2 | CYC1 | 2 | GO:0005739 GO:0016020 |
| SLC27A4 | MTX1 | 2 | GO:0016020 GO:0016021 |
| RPS6KC1 | BAMBI | 2 | GO:0016020 GO:0005737 |
| GPR143 | GPSM3 | 2 | GO:0005737 GO:0007165 |
| GHRHR | MLNR | 2 | GO:0005886 GO:0007186 |
| NR6A1 | HESX1 | 2 | GO:0006355 GO:0005634 |
| TMED1 | PCDH1 | 2 | GO:0005886 GO:0007267 |
| MAML2 | WDR12 | 2 | GO:0007219 GO:0005634 |
| MEOX1 | SOX10 | 2 | GO:0045944 GO:0005634 |
| PIP5K3 | TM9SF2 | 2 | GO:0016020 GO:0005768 |
| MKL1 | NKX2-3 | 2 | GO:0005634 GO:0006355 |
| RRM2B | ZNF148 | 1 | GO:0005634 |
| RRM2 | CARM1 | 1 | GO:0005737 |
| RRM2B | GNL3 | 1 | GO:0005634 |
| RRM2 | EEF2 | 1 | GO:0005737 |
| RRM2B | EEF2 | 1 | GO:0005737 |
| RRM2 | IFI16 | 1 | GO:0005737 |
| SPHK1 | SOX30 | 1 | GO:0005737 |
| SMTN | MYL1 | 1 | GO:0007517 |
| SMTN | PCYT1B | 1 | GO:0005737 |
| SRP68 | PDE5A | 1 | GO:0005737 |
| GLUD1 | MDH1 | 1 | GO:0055114 |
| ATPAF2 | ATPAF1 | 1 | GO:0005739 |
| CD84 | SLAMF7 | 1 | GO:0007155 |
| BFSP1 | UPP2 | 1 | GO:0005737 |
| SLU7 | ZBTB5 | 1 | GO:0005634 |
| PACAP | GOLGA3 | 1 | GO:0005737 |
| SLU7 | ZNF337 | 1 | GO:0005634 |
| SLU7 | KIAA1539 | 1 | GO:0005634 |
| ACTL6B | FOXP1 | 1 | GO:0005634 |
| SLC7A11 | FAM57A | 1 | GO:0016021 |
| SLC7A8 | FAM57A | 1 | GO:0005886 |
| TP53RK | PCGF1 | 1 | GO:0005634 |
| POP5 | POP1 | 1 | GO:0005634 |
| PCYT1B | MIB2 | 1 | GO:0005737 |
| RPS6KC1 | NUDT3 | 1 | GO:0005737 |
| PCYT1B | DVL1L1 | 1 | GO:0005737 |
| ROM1 | CNGB1 | 1 | GO:0016020 |
| HAAO | GAD2 | 1 | GO:0005737 |
| GUCA2B | GUCA1B | 1 | GO:0007589 |
| TMED1 | PRDM4 | 1 | GO:0007165 |
| IL11 | C18ORF54 | 1 | GO:0005576 |
| MAML2 | DLL4 | 1 | GO:0007219 |
| NPFF | NPY5R | 1 | GO:0007268 |
| PAN3 | PAIP1 | 1 | GO:0005737 |
| PAN3 | PAIP2 | 1 | GO:0005737 |
| SLC7A8 | BAAT | 1 | GO:0005737 |
| BFSP1 | UPP1 | 1 | GO:0005737 |
| BFSP1 | OSBP2 | 1 | GO:0016020 |
| PHLDB2 | SGCG | 1 | GO:0005737 |
