# Supplementary material for: Geometric De-noising of Protein-Protein Interaction Networks
Source: PLoS Comput Biol. 2009 Aug 7;5(8):e1000454. doi: 10.1371/journal.pcbi.1000454 (PMC2711306; doi:10.1371/journal.pcbi.1000454)
Supplement: Table S4 — Protein-protein interaction predictions where both proteins in the pair share at least one GO term corresponding to the “biological process”. (0.07 MB DOC) [file pcbi.1000454.s005.doc]

Table S4: Protein-protein interaction predictions where both proteins in the pair share at least one GO term corresponding to the ``biological process’’. In this analysis we take into account only those protein pairs in which both proteins are annotated with at least one GO term which is not a root term (GO:0008150 for biological process).

| **Official Symbol A** | **Official Symbol B** | **Number of shared terms** | **Shared GO terms** |
| --- | --- | --- | --- |
| POU3F2 | POU3F3 | 9 | GO:0021799 GO:0021799 GO:0021799 GO:0021869 GO:0021869 GO:0021869 GO:0045944 GO:0045944 GO:0045944 |
| SIM1 | SIM2 | 5 | GO:0006355 GO:0030154 GO:0007165 GO:0007275 GO:0007399 |
| CCL20 | XCL1 | 4 | GO:0007165 GO:0007267 GO:0006935 GO:0006955 |
| SLC7A5 | SLC7A8 | 4 | GO:0006520 GO:0006810 GO:0015804 GO:0015807 |
| MAML2 | MAML3 | 4 | GO:0045944 GO:0007219 GO:0006350 GO:0006355 |
| CCL19 | CCL25 | 3 | GO:0006935 GO:0006954 GO:0006955 |
| CCL20 | XCR1 | 3 | GO:0007165 GO:0006935 GO:0006954 |
| CRHBP | CRH | 3 | GO:0007565 GO:0007611 GO:0007165 |
| DAZ1 | DAZAP1 | 3 | GO:0030154 GO:0007275 GO:0007283 |
| CCR6 | XCL2 | 2 | GO:0007165 GO:0006935 |
| SLC7A11 | SLC7A7 | 2 | GO:0006810 GO:0006865 |
| SLC7A10 | SLC7A8 | 2 | GO:0006810 GO:0015804 |
| SLC7A7 | SLC7A8 | 2 | GO:0006520 GO:0006810 |
| UTY | UTX | 2 | GO:0055114 GO:0016568 |
| RRM2B | PLAGL1 | 2 | GO:0006917 GO:0006917 |
| TESK1 | TESK2 | 2 | GO:0006468 GO:0007283 |
| CHRNA2 | CHRNA5 | 2 | GO:0007165 GO:0006811 |
| CHRNA2 | CHRNA3 | 2 | GO:0007165 GO:0006811 |
| MED18 | MED8 | 2 | GO:0006350 GO:0006355 |
| MKL1 | ETV4 | 2 | GO:0045941 GO:0006355 |
| GP5 | GP9 | 2 | GO:0007596 GO:0007155 |
| SIM2 | AHRR | 2 | GO:0006355 GO:0007165 |
| NR6A1 | JMJD2A | 2 | GO:0006355 GO:0006350 |
| NR6A1 | NR1D1 | 2 | GO:0006355 GO:0006350 |
| NR6A1 | NR1D2 | 2 | GO:0006355 GO:0006350 |
| PRIMA1 | COLQ | 1 | GO:0042135 |
| SLC7A11 | SLC7A10 | 1 | GO:0006810 |
| SLC7A11 | SLC7A5 | 1 | GO:0006810 |
| SLC7A11 | SLC3A1 | 1 | GO:0006810 |
| RRM2 | WWOX | 1 | GO:0055114 |
| RRM2B | WWOX | 1 | GO:0055114 |
| SMTN | MYL1 | 1 | GO:0007517 |
| GLUD1 | MDH1 | 1 | GO:0055114 |
| CDH18 | CDH19 | 1 | GO:0007156 |
| RPP21 | RPP14 | 1 | GO:0008033 |
| POP4 | RPP14 | 1 | GO:0008033 |
| RPP30 | RPP25 | 1 | GO:0008033 |
| RPP38 | RPP25 | 1 | GO:0008033 |
| SSTR4 | MRGPRX2 | 1 | GO:0007165 |
| CD84 | SLAMF7 | 1 | GO:0007155 |
| MED31 | MED8 | 1 | GO:0006355 |
| EAF1 | EAF2 | 1 | GO:0006355 |
| PDCD6 | ALG2 | 1 | GO:0051592 |
| GPR143 | GPSM3 | 1 | GO:0007165 |
| GHRHR | MLNR | 1 | GO:0007186 |
| GUCA2B | GUCA1B | 1 | GO:0007589 |
| NR6A1 | HESX1 | 1 | GO:0006355 |
| SLC25A17 | ABCD2 | 1 | GO:0006810 |
| TMED1 | PCDH1 | 1 | GO:0007267 |
| TMED1 | PRDM4 | 1 | GO:0007165 |
| MAML2 | WDR12 | 1 | GO:0007219 |
| MAML2 | DLL4 | 1 | GO:0007219 |
| NPFF | NPY5R | 1 | GO:0007268 |
| MEOX1 | SOX10 | 1 | GO:0045944 |
| MKL1 | NKX2-3 | 1 | GO:0006355 |
