# Supplementary material for: Geometric De-noising of Protein-Protein Interaction Networks
Source: PLoS Comput Biol. 2009 Aug 7;5(8):e1000454. doi: 10.1371/journal.pcbi.1000454 (PMC2711306; doi:10.1371/journal.pcbi.1000454)
Supplement: Table S5 — Protein-protein interaction predictions where both proteins participate in the same KEGG pathway. (0.04 MB DOC) [file pcbi.1000454.s006.doc]

**Table S5: Protein-protein interaction predictions where both proteins participate in the same KEGG pathway. Number of shared GO terms refers to the ``biological process’’ or ``cellular component ’’ terms (see Table S3 for details).**

| **Official Symbol A** | **Official Symbol B** | **KEGG pathway** | **Pathway description (KEGG Orthology)** | **Number of shared GO terms** |
| --- | --- | --- | --- | --- |
| COX4I2 | CYC1 | hsa00190; hsa05010; hsa05012 | Oxidative phosphorylation; Alzheimer's disease; Parkinson's disease | 2 |
| GP5 | GP9 | hsa04512; hsa04640 | ECM-receptor interaction; Hematopoietic cell lineage | 7 |
| CCL19 | CCL25 | hsa04060 | Cytokine-cytokine receptor interaction | 5 |
| CCL20 | XCL1 | hsa04060 | Cytokine-cytokine receptor interaction | 6 |
| CCL20 | XCR1 | hsa04060 | Cytokine-cytokine receptor interaction | 3 |
| CCR6 | XCL2 | hsa04060 | Cytokine-cytokine receptor interaction | 2 |
| TNFRSF18 | TNFRSF4 | hsa04060 | Cytokine-cytokine receptor interaction | 0 |
| TNFRSF4 | TNFRSF9 | hsa04060 | Cytokine-cytokine receptor interaction | 2 |
| GHRHR | MLNR | hsa04080 | Neuroactive ligand-receptor interaction | 2 |
| HIST2H4 | HIST1H2AE | hsa05322 | Systemic lupus erythematosus | 0 |
| MAML2 | MAML3 | hsa04330 | Notch signaling pathway | 6 |
| MAML2 | DLL4 | hsa04330 | Notch signaling pathway | 4 |
