# Supplementary material for: Geometric De-noising of Protein-Protein Interaction Networks
Source: PLoS Comput Biol. 2009 Aug 7;5(8):e1000454. doi: 10.1371/journal.pcbi.1000454 (PMC2711306; doi:10.1371/journal.pcbi.1000454)
Supplement: Table S6 — Predicted false positives. (1.10 MB DOC) [file pcbi.1000454.s007.doc]

**Table S6: Predicted false positives. These edges from ``HumanBG’’ network correspond to pairs of proteins which are substantially further away than most of the edges. Also these pairs of proteins either: (i) do not share common ``cellular localization’’ GO terms, (ii) have at least one protein not annotated with ``cellular localization’’ GO term.**

| **Official Symbol A** | **Official Symbol B** |
| --- | --- |
| FGF2 | CSNK2A1 |
| FGF2 | CSNK2A2 |
| FGF2 | FGFR1 |
| ACTA1 | TRIP6 |
| CTNNB1 | PTPN1 |
| ERBB2 | HSPCA |
| CDC42 | PAK6 |
| TOLLIP | TLR4 |
| TOLLIP | TLR2 |
| TOLLIP | IL1RAP |
| CLTC | TFE3 |
| NOTCH1 | NOV |
| XRCC6 | CD40 |
| XRCC6 | CLU |
| UBE1 | HSPH1 |
| INS | MLL |
| INS | A.2 |
| TAPBP | COPB |
| COPG2 | COPB |
| COPG2 | DRD1 |
| DAXX | FASLG |
| TGFB1 | TGFBR1 |
| TGFBR2 | DAB2 |
| FAS | SUMO1 |
| FAS | UBE2I |
| FAS | FBF1 |
| FASLG | SUMO1 |
| FASLG | FNBP3 |
| UBE2I | TNFRSF1A |
| UBE2I | SLC2A1 |
| UBE2I | HGS |
| UBE2I | MAP3K1 |
| UBE2I | C14ORF32 |
| SUMO1 | TNFRSF1A |
| HSPB1 | ACTC |
| MCRS1 | CSNK2B |
| MCRS1 | PRKAR1A |
| AR | SRC |
| AR | RAF1 |
| AR | CAV1 |
| MAP3K5 | TXN |
| AGER | S100P |
| AGER | ELP3 |
| AGER | S100A1 |
| HMGB1 | CSNK1A1 |
| HMGB1 | HNRPK |
| HMGB1 | PLG |
| HMGB1 | PLAT |
| RNF5 | UBE2D3 |
| UBE2D3 | NEDD4 |
| UBE2D3 | RNF126 |
| BAT5 | NIFIE14 |
| PBX2 | BAIAP2 |
| NUP62 | GORASP2 |
| HSPA1A | BAG3 |
| STUB1 | HSPCA |
| STUB1 | UBE2D1 |
| DNAJA3 | RASA1 |
| DNAJA3 | FLJ36766 |
| DNAJA3 | SMAD9 |
| TP53 | EFEMP2 |
| TP53 | TAF1 |
| BAT3 | EFEMP2 |
| BAT3 | ZFYVE9 |
| BAT3 | KLHL12 |
| HSF1 | REPS1 |
| IKBKG | APG16L |
| IKBKG | UBB |
| APOB | HSPCA |
| BAG1 | PDGFRB |
| BAG1 | HBEGF |
| BAG3 | DAZAP2 |
| PTMA | VIPR1 |
| CDK9 | IL6ST |
| TRIM39 | RP42 |
| TRIM39 | PDLIM5 |
| SPOP | DOK2 |
| SPOP | GDI1 |
| NRIP1 | DIPA |
| NCOA3 | BMP6 |
| NCOA3 | BMP7 |
| EXOSC4 | MPZL1 |
| EXOSC4 | C1ORF19 |
| HRAS | TTC1 |
| HRAS | IRAK2 |
| HRAS | ZNFN1A3 |
| RAP1GA1 | GNAZ |
| COBRA1 | TH1L |
| TH1L | ARAF |
| BAT8 | C10ORF12 |
| BAT8 | KLF6 |
| BAT8 | LMO2 |
| BAT8 | E4F1 |
| FLJ32855 | KIAA0980 |
| FLJ32855 | PLSCR1 |
| FLJ32855 | HGS |
| LNX | DDX17 |
| LNX | MAGEA11 |
| LNX | DVL3 |
| LNX | MGC33889 |
| LNX | RABAC1 |
| HLA-G | LILRB2 |
| COPB | CDKN2A |
| COPB | PDGFRB |
| COPB | TRIM37 |
| COPB | KDELR1 |
| COPB | COPG |
| LILRB2 | HLA-F |
| GRB2 | HD |
| GRB2 | HNRPC |
| GRB2 | MAPK14 |
| GRB2 | ELK1 |
| GRB2 | DAB2 |
| GRB2 | MTA1 |
| GRB7 | SFN |
| NGB | GNAO1 |
| ARGBP2 | VCL |
| MDM2 | NUMB |
| ATM | AP2A2 |
| POLR2A | CCNK |
| POLR2A | CSH2 |
| TUBB | NCOA6 |
| SDC3 | CUTL1 |
| SDC3 | COL5A1 |
| TBCD | ARL2 |
| TGM2 | HD |
| TGM2 | FN1 |
| TGM2 | SPARC |
| NCALD | DTX2 |
| PLK1 | BRCA2 |
| PLK1 | CHEK2 |
| PLK1 | PSMB1 |
| PLK1 | PSMB4 |
| HLA-B | TRIM28 |
| HLA-B | PAICS |
| RUVBL1 | ACTL6A |
| RUVBL1 | PLG |
| TRA@ | CD3E |
| TRA@ | LEF1 |
| TRA@ | TRAT1 |
| TRA@ | CD3Z |
| VIL2 | PIK3R1 |
| VIL2 | MAPK8 |
| VIL2 | SYK |
| VIL2 | PTK2 |
| TRIM28 | TNFRSF10D |
| MAGEA1 | HLA-A |
| PAICS | EPB41 |
| PAICS | GNAS |
| PAICS | ITGB4BP |
| PFAS | TFE3 |
| VARS2 | TFE3 |
| KRTAP4-12 | PDGFRB |
| KRTAP4-12 | HHEX |
| KRTAP4-12 | FBXW5 |
| HLA-A | UGCGL1 |
| PDIA3 | STAT3 |
| MAGEA4 | RNF36 |
| MAGEA4 | MGC27019 |
| NFKBIL1 | MRPS18B |
| TNFRSF14 | P4HB |
| TNFRSF14 | TNFSF12-TNFSF13 |
| TNFRSF1A | TRAF2 |
| TNFRSF1A | PTPN11 |
| TNFRSF1A | FANCD2 |
| TNFRSF1A | STAT1 |
| RFP | PML |
| RFP | PRAM-1 |
| RFP | FBF1 |
| KIAA1446 | ZNF417 |
| KIAA1446 | DLG1 |
| KIAA1446 | RBL1 |
| KIAA1446 | DLG4 |
| ZBTB16 | DIPA |
| FBF1 | PEPP-2 |
| FBF1 | HIF1AN |
| SIAH1 | DAB1 |
| MYC | K-ALPHA-1 |
| MYC | HNRPC |
| MYC | RAF1 |
| MYC | ACTL6A |
| MYC | TIAM1 |
| JTV1 | EPRS |
| JTV1 | FUBP1 |
| JTV1 | PARK2 |
| JTV1 | EPS8 |
| JTV1 | DARS |
| MGC11271 | VCL |
| MGC11271 | GPS2 |
| MGC11271 | C19ORF25 |
| EPRS | TFE3 |
| LMO3 | HNRPM |
| VDP | PLCG1 |
| VDP | PTP4A3 |
| VDP | MGC15882 |
| NSF | SAP18 |
| HDAC2 | CDC20 |
| SRC | SRF |
| SRC | PELP1 |
| SRC | HSPCA |
| SRC | FOXO1A |
| SRC | NCOA6 |
| SRC | MATK |
| SRC | PKD1 |
| SRC | STAT1 |
| SRC | SMARCB1 |
| GAB2 | AKT1 |
| DLG4 | DLC2 |
| GAB1 | MAP3K3 |
| GRIN2A | DNCL1 |
| NTRK1 | RGS19IP1 |
| NTRK1 | NGFB |
| PIK3R1 | TGFBR1 |
| BCAR1 | PKD1 |
| FYN | SCAP2 |
| FYN | BCL3 |
| SF3B4 | BMPR1A |
| EFEMP2 | RIBC2 |
| EFEMP2 | SGTA |
| EFEMP2 | PLSCR1 |
| EFEMP2 | UBQLN1 |
| EFEMP2 | TU3A |
| NFKBIA | DNCL1 |
| PXN | GIT2 |
| PXN | ARHGEF7 |
| SNAP91 | OGT |
| PC4 | CENTA1 |
| HD | FNBP3 |
| HD | TCERG1 |
| HD | CBS |
| CHP | SLC9A1 |
| SIAHBP1 | SYNCRIP |
| SIAHBP1 | DDX3X |
| SIAHBP1 | HNRPL |
| SIAHBP1 | PABPC1 |
| SF3A3 | CENTA1 |
| SF3B1 | APG12L |
| TTN | ANKRD2 |
| UGP2 | ARIH2 |
| SYNCRIP | ZNF9 |
| RNMTL1 | CUL2 |
| DDX3X | DNCL1 |
| IMP-1 | PRKRA |
| G3BP | RASA1 |
| EWSR1 | ACTL6A |
| EWSR1 | MGC2749 |
| EWSR1 | TAF1 |
| HNRPC | DIPA |
| HNRPC | FXR2 |
| HNRPC | PDGFB |
| KHSRP | ACF |
| P4HB | UBQLN1 |
| P4HB | ELF3 |
| P4HB | TRIP6 |
| STOM | CUL2 |
| KIAA0980 | RIBC2 |
| KIAA0980 | JUNB |
| KIAA0980 | RGS2 |
| KIAA0980 | TU3A |
| MGC17403 | MAGEA11 |
| RIBC2 | AP2B1 |
| GCC1 | FLJ12529 |
| MBIP | RAP1GDS1 |
| ZNF426 | LPXN |
| MGC10471 | ZNF24 |
| LZTS2 | ZNF581 |
| PPP1CA | TGFBR1 |
| SMG1 | RENT1 |
| CDH5 | VIM |
| PARC | UBE2G2 |
| UBE2G2 | CORO1A |
| UBE2G2 | USP15 |
| UBE2G2 | AUP1 |
| NEDD4 | DAZAP2 |
| NEDD4 | SGK |
| CABIN1 | AMPH |
| CABIN1 | PPP3CC |
| CABIN1 | PPP3CB |
| CABIN1 | DNM1 |
| HOM-TES-103 | XRCC4 |
| RNF183 | FBP1 |
| FOS | MAP3K7 |
| ESR1 | MKNK2 |
| PSCD2 | JM11 |
| PSCD2 | TRIM23 |
| APPL | DCC |
| BRDG1 | POLR2E |
| RUVBL2 | ACTL6A |
| RUVBL2 | SSSCA1 |
| 76P | AP2B1 |
| 76P | GFAP |
| 76P | MAD1L1 |
| USHBP1 | FANCG |
| USHBP1 | MED28 |
| USHBP1 | N/A |
| USHBP1 | KNTC2 |
| PAK7 | RPA3 |
| PAK7 | S100A7 |
| PAK7 | HSPC128 |
| PAK7 | S100A9 |
| HK3 | IGFBP4 |
| HK3 | LEP |
| ACF | TNPO2 |
| ACF | APOBEC1 |
| BTG2 | PRKCABP |
| LYN | CSNK2B |
| LYN | SCAP2 |
| HCK | SCAP2 |
| RGS19 | RGS19IP1 |
| RAF1 | RB1 |
| RAF1 | RBL2 |
| RAF1 | PDGFRB |
| ZAK | SFN |
| RANBP9 | MET |
| BIRC4 | DLC2 |
| RPA3 | ZNF9 |
| RB1CC1 | PREI3 |
| RB1CC1 | DBNL |
| PINX1 | CALM3 |
| RPL31 | PSTPIP1 |
| RPL31 | YWHAZ |
| RPS3A | GC20 |
| TXN | COL1A1 |
| CALM3 | RIOK3 |
| CALM3 | CALD1 |
| CALM3 | CAMK2A |
| CALM3 | RAB3B |
| RP11-301I17.1 | SULT1E1 |
| NIF3L1 | DMRTB1 |
| NIF3L1 | CPSF5 |
| RAB22A | EEA1 |
| STAP2 | BMP4 |
| LYAR | RPL21 |
| LYAR | FLJ10534 |
| LYAR | EIF3S2 |
| RPS9 | CENTA1 |
| FLJ10534 | BMPR2 |
| FLJ10534 | SMNDC1 |
| FLJ10534 | GC20 |
| DDX21 | PSTPIP1 |
| EIF3S2 | EXOSC6 |
| KRTHA1 | DNPEP |
| KRTHA1 | CDC20 |
| KRTHB2 | DNPEP |
| KRTHB5 | DLG3 |
| PTK9L | CAPZA1 |
| PTK9L | KRTHA6 |
| FLJ10324 | PEPP-2 |
| FLJ10324 | ATIC |
| PEPP-2 | SFRS16 |
| PEPP-2 | RBM9 |
| CREBBP | CSNK2A2 |
| SELS | SAA1 |
| HSPA4 | TTC1 |
| N/A | FKBP1A |
| N/A | NUP37 |
| N/A | VIM |
| C17ORF28 | RCN3 |
| DLG1 | KIF1B |
| DLG1 | CALM2 |
| C16ORF48 | PLSCR1 |
| C16ORF48 | PDLIM7 |
| C16ORF48 | MDFI |
| AFAP | ACTC |
| ACTC | PTK9 |
| ACTC | LASP1 |
| ACTC | MYLK |
| ACTC | WAS |
| PRKCB1 | BMPR2 |
| PRKCB1 | GSK3B |
| PRKCD | PLSCR3 |
| PRKCE | COPB2 |
| PRKCE | GNA13 |
| PRKCE | KRT1 |
| SNIP | SNAP25 |
| FLNA | LNK |
| LSM8 | ITSN2 |
| LOC84661 | ASH2L |
| STAT3 | TSHR |
| STAT5A | PPIB |
| TIFA | IRAK1 |
| TRAF2 | CAV1 |
| TRAF2 | RAD23A |
| TRAF2 | MAPK9 |
| TRAF2 | CD40 |
| TRAF2 | BANP |
| IRAK1 | ITGAM |
| MAP2K1 | TRAF3 |
| RALA | REPS1 |
| RAP1A | KRIT1 |
| TRAF6 | UBB |
| TRAF6 | IRAK2 |
| PRKAR2A | RUNX1T1 |
| PRKAR2A | CBFA2T3 |
| RGS4 | COPB2 |
| ERBB3 | TEBP |
| ERBB3 | PA2G4 |
| GNAI1 | RGS14 |
| GNAI2 | TTC1 |
| GNAI2 | IGF1R |
| GNAO1 | ADRA2C |
| RGS7 | SNAPAP |
| SNAPAP | TRPV1 |
| GNAI3 | RGS14 |
| CD47 | BNIP3 |
| CD47 | THBS1 |
| CD47 | UBQLN1 |
| SP1 | CD2 |
| SIN3A | RNF12 |
| IRF4 | 25181 |
| JUND | MAPK9 |
| CALM1 | CALD1 |
| RIT2 | POU4F1 |
| VCL | FEN1 |
| VCL | NONO |
| VCL | HNRPK |
| VCL | MYL6 |
| CRADD | LRDD |
| RNF11 | RPS27A |
| RNF11 | ZNF350 |
| RNF11 | ZFYVE9 |
| RNF11 | CBLB |
| RPS27A | DAZAP2 |
| RABGEF1 | MGC2749 |
| RNF14 | SMURF1 |
| RNF2 | TFCP2 |
| HIST2H2BE | HSPD1 |
| ACTN4 | CAMK2G |
| ACTN4 | USP6NL |
| HIP2 | DNCL1 |
| HIP2 | NEDD8 |
| HIP2 | RIPK5 |
| RNF7 | CUL2 |
| CSNK2B | RNPS1 |
| CSNK2B | CSNK2A2 |
| CUL2 | TFE3 |
| CUL2 | TIMM13 |
| CUL2 | KCTD5 |
| CUL2 | DLG3 |
| DIPA | CDKN1A |
| DIPA | KRT18 |
| DIPA | SMARCD1 |
| DIPA | TCFL1 |
| DIPA | NEK6 |
| DIPA | EZH2 |
| DIPA | MGC13138 |
| DIPA | HMG20B |
| DIPA | EPS8 |
| DIPA | KRT17 |
| DIPA | ZNF165 |
| RNPS1 | CSNK2A2 |
| RNPS1 | LUC7L2 |
| RNPS1 | AP2B1 |
| SF3B3 | CFLAR |
| SFRS1 | SNRP70 |
| SFRS1 | YT521 |
| SFRS1 | C1QBP |
| SFRS2 | YT521 |
| SNRP70 | SNRPC |
| SNRP70 | CRP |
| SNRP70 | CPSF5 |
| CPSF5 | CPSF6 |
| CPSF5 | FLJ12529 |
| CPSF6 | WWP2 |
| CPSF6 | NCK2 |
| CPSF6 | KLHL12 |
| C20ORF14 | ARAF |
| C20ORF14 | CD2BP2 |
| FNBP3 | PIAS3 |
| FNBP3 | HIPK1 |
| FNBP3 | PRKAR1A |
| FNBP3 | FNBP4 |
| PRPF8 | CD2BP2 |
| CSNK2A2 | MAPK14 |
| CSNK2A2 | TGFBR1 |
| CSNK2A2 | NCL |
| CSNK2A2 | HSPCA |
| CSNK2A2 | TRA1 |
| CSNK2A2 | PIN1 |
| CSNK2A2 | PAK1 |
| CSNK2A2 | PTEN |
| STRAP | GH1 |
| U5-116KD | CD2BP2 |
| ASCC3L1 | YWHAG |
| ASCC3L1 | CD2BP2 |
| ASCC3L1 | SMNDC1 |
| AP2B1 | MLH1 |
| AP2B1 | KIAA0408 |
| AP2B1 | TERF2IP |
| AP2B1 | THAP1 |
| PSME3 | SERF2 |
| PSME3 | MCC |
| PSME3 | ITPKB |
| MAGI1 | SFN |
| MAGI1 | FCHSD2 |
| RNU11 | SNRPD2 |
| RNU11 | SNRPG |
| RNU11 | B.1 |
| SNRPD2 | RNU12 |
| SNRPD2 | SMN2 |
| SNRPG | RNU12 |
| U2AF1 | ZNF265 |
| B.1 | RNU12 |
| B.1 | AKAP3 |
| BCR | ERCC3 |
| BCR | RB1 |
| BCR | BMPR1B |
| BCR | TGFBR1 |
| BCR | NCK2 |
| SHC1 | NDP52 |
| TLN1 | NRAP |
| FES | PSMD13 |
| ABL1 | C3 |
| RB1 | GNB2L1 |
| RHOC | VHL |
| STK16 | RPIA |
| STK16 | TSRC1 |
| STK16 | NAGK |
| STK16 | ELK1 |
| STK16 | TGIF |
| RDS | PRPH |
| MAGED1 | PJA1 |
| NME1 | STK6 |
| NME2 | LYZ |
| PPARBP | MED28 |
| PCNA | ANXA2 |
| PDE6D | RAD23A |
| PDE6D | RAB13 |
| RPH3AL | RAB8A |
| ZYX | LATS1 |
| RAB3A | RABIF |
| RAB3B | CALM2 |
| RAB3D | CHM |
| C6ORF55 | RABAC1 |
| C6ORF55 | CDK4 |
| FLJ10204 | RABAC1 |
| FLJ10204 | MDFI |
| NOS1 | DNCL1 |
| PTPN11 | SELE |
| RPL11 | BLMH |
| PML | MAPK14 |
| PHLDA1 | EIF3S7 |
| NTRK2 | DNCL1 |
| C3 | CR2 |
| RPN1 | UBQLN2 |
| RAD23A | PSMD4 |
| RPS14 | TAF9 |
| CNNM3 | HNRPK |
| MPP6 | KCNJ12 |
| DAZAP2 | ZFYVE9 |
| DAZAP2 | PLSCR1 |
| DAZAP2 | UBB |
| DAZAP2 | LMO2 |
| DAZAP2 | STAM2 |
| DAZAP2 | AATF |
| DDIT3 | PICALM |
| DDIT3 | DHRS10 |
| CREB1 | CSEN |
| MAPK14 | ATF2 |
| MAPK14 | SMAD7 |
| MAPK14 | MKNK2 |
| MAPK14 | MAP2K3 |
| MAPK14 | MARS |
| MAPK14 | MEF2A |
| MAPK14 | DUSP10 |
| TRAF4 | SIGLEC7 |
| COL1A1 | DDR2 |
| COL2A1 | ITGA2B |
| COL2A1 | DDR1 |
| ELN | ASS |
| ARAF | COPS3 |
| ARAF | KLHL12 |
| ARAF | EFEMP1 |
| ARAF | PBK |
| DCTN1 | GSTK1 |
| RTN1 | DKFZP566M1046 |
| PLEKHF2 | MAX |
| PLEKHF2 | CHIC2 |
| PLEKHF2 | PRKAR1A |
| PLEKHF2 | SP192 |
| PLEKHF2 | TSC22D3 |
| RTN4 | LRCH4 |
| ACTL6A | SMARCA2 |
| ACTL6A | SMARCA4 |
| FDFT1 | PSTPIP1 |
| THRAP4 | MED28 |
| CTSL | KNG1 |
| CTSL | COL18A1 |
| ITGB3BP | ITGB5 |
| ITGB3BP | ITGB3 |
| THRB | TRIP6 |
| FADD | DAP3 |
| EPHB2 | ACP1 |
| HOMER1 | CENTG1 |
| FKBP1A | DHFR |
| RYR2 | FKBP1B |
| RYR2 | SRI |
| ANXA6 | CR2 |
| S100A10 | TRPV6 |
| S100A10 | PLG |
| RAB6A | RABGAP1 |
| S100A13 | FGF1 |
| FGF1 | FGFR3 |
| FGF1 | FGFR1 |
| S100A6 | PRR6 |
| COPS5 | MIF |
| COPS5 | RNF139 |
| S100A8 | USF2 |
| S100A8 | ASB3 |
| S100A9 | ASB3 |
| CAPN1 | INPP4A |
| BIK | BCL2A1 |
| BCL2A1 | HRK |
| BCL2A1 | BAK1 |
| BCL2L1 | IRS2 |
| BCL2L1 | BCAP31 |
| COL4A3 | COL4A3BP |
| BIN1 | ITGA3 |
| CHD1 | ARRB1 |
| SAFB2 | SCAM-1 |
| SALL2 | DKFZP761I2123 |
| SALL2 | C18ORF9 |
| SAP18 | KIAA1279 |
| SAP18 | SUFU |
| ZNFN1A1 | AP2M1 |
| KIAA1279 | DOK2 |
| EIF3S6 | BAT2 |
| EIF3S6 | SP192 |
| SUFU | MGC13138 |
| SAT | HOXB6 |
| TSC22D4 | CDC23 |
| DNPEP | KRTHA3A |
| GAPDH | PLD2 |
| ITSN1 | TSG101 |
| BLK | UBE3A |
| UBE3A | UBE2G1 |
| RGS20 | TXNL2 |
| TP53BP1 | DNCL1 |
| MLH1 | TRIM29 |
| RAD51 | CAB39 |
| RAD51 | RAD54B |
| SMC1L1 | CASP4 |
| SNTA1 | MAST1 |
| SNTA1 | KCNJ12 |
| SCNN1A | WWP2 |
| SCNN1B | NEDD4L |
| SCNN1G | NEDD4L |
| STX1A | SLC6A4 |
| WWP2 | TGFBR1 |
| WWP2 | PDLIM7 |
| WWP2 | SF1 |
| WWP2 | VASP |
| CCR5 | CCL3 |
| CSPG2 | CCL5 |
| CSPG2 | FBLN2 |
| IGFBP7 | VPS24 |
| CCL3 | CCR4 |
| SDC1 | LACRT |
| SDC1 | IL8 |
| PIGR | SGK |
| CXCL5 | IL8RA |
| CXCL5 | IL8RB |
| IL8RA | CXCL2 |
| IL8RB | CXCL2 |
| MMP9 | CD44 |
| CASK | FCHSD2 |
| FN1 | PKD1 |
| FN1 | VHL |
| RGS19IP1 | SH3BP4 |
| RGS19IP1 | KIF1B |
| RGS19IP1 | TGFBR3 |
| RGS19IP1 | ITGA6 |
| RGS19IP1 | MCM7 |
| RGS19IP1 | NUP93 |
| RGS19IP1 | DRD3 |
| GRM3 | PPM1A |
| RAB5A | RAB37 |
| CXCL12 | CXCR4 |
| CD4 | BTRC |
| CD4 | SPG21 |
| CD4 | CTSD |
| MMP2 | BACE1 |
| BMPR2 | FRS3 |
| BMPR2 | CTBP1 |
| BMPR2 | HNRPR |
| BMPR2 | LIMK1 |
| ACVR1 | STAT1 |
| ACVR1 | SMAD1 |
| ACVR1 | FKBP4 |
| FLJ22494 | DUT |
| ARF6 | DDEF2 |
| ARF6 | DHPS |
| SEC13L1 | NUP133 |
| SEC13L1 | NUP160 |
| CD7 | LGALS1 |
| BMPR1A | ZMYND11 |
| BMPR1A | MAP3K7IP1 |
| ZFYVE9 | SOC |
| ZFYVE9 | ETS2 |
| TRIP13 | ADAM15 |
| TRIP13 | LOXL4 |
| TRIP13 | MAWBP |
| TRIP13 | PLK3 |
| SELP | AP1M1 |
| SEMA4D | PLXNB1 |
| PTPRC | LGALS1 |
| PTPRC | GANAB |
| ACVR2A | SYNJ2BP |
| RBPMS | GRAP2 |
| RBPMS | PIN1 |
| RBPMS | CDC23 |
| RBPMS | RBM9 |
| RBPMS | HOXA9 |
| RBPMS | HEYL |
| SMAD1 | INPP4A |
| CDK5 | CABLES2 |
| CDK5R1 | CDK5RAP3 |
| HIST3H3 | TRPM7 |
| SNIP1 | DVL2 |
| SF3A1 | SMURF1 |
| SF3A1 | APG12L |
| BMPR1B | FBXW5 |
| RAB6B | RAB6IP2 |
| RAB6B | GTP |
| PLEKHB1 | GNAS |
| FBXW5 | TGFBR1 |
| FBXW5 | MDFI |
| RRAS2 | TRAF3 |
| ARHGEF6 | PAK3 |
| APG16L | APG12L |
| RHOJ | WAS |
| CDK4 | TGFBR1 |
| MKNK2 | ESR2 |
| MKNK2 | MAPK1 |
| TGFBR1 | SMAD2 |
| TGFBR1 | DAB2 |
| TGFBR1 | PIK3R2 |
| TGFBR1 | CDK6 |
| TGFBR1 | IKBKB |
| TGFBR1 | NEK6 |
| TGFBR1 | CSNK1D |
| TGFBR1 | CHUK |
| TGFBR1 | STAT1 |
| MAP2K3 | MAP3K4 |
| MAP2K3 | MAPK8IP2 |
| MAP2K3 | MAPK3 |
| SS18L1 | RFXDC1 |
| SFN | KIAA0408 |
| SFN | KIAA0514 |
| PLK4 | TEC |
| PLK4 | KIAA0774 |
| USP8 | OTUB1 |
| WEE1 | CDCA3 |
| YWHAG | KIAA0408 |
| YWHAG | FOXO1A |
| ITCH | OCLN |
| PAK4 | ITGAV |
| CDC2 | DAB2 |
| ZNF638 | PLSCR1 |
| RHPN2 | TCF1 |
| KIAA0408 | MAGEA11 |
| KIAA0408 | MDFI |
| KIAA0514 | MDFI |
| HNRPU | SYK |
| APLP2 | DAB1 |
| APLP2 | APBB3 |
| IRS1 | MAPK9 |
| IRS2 | UBTF |
| KNS2 | APP |
| KRT18 | PKD1 |
| KRT18 | MCC |
| PKP3 | DSC3 |
| ABLIM1 | KCNJ12 |
| NONO | FXR2 |
| WNT1 | PORCN |
| ACVR1B | SNX2 |
| HNRPR | HRMT1L2 |
| YT521 | KHDRBS3 |
| YT521 | EMD |
| FUS | PTBP1 |
| FUS | ITGB4BP |
| RBM7 | HNRPK |
| CDC2L6 | MYCBPAP |
| STAT1 | IL2RB |
| STAT1 | MDK |
| PTK2 | APG12L |
| ACHE | LGTN |
| SGK | SLC9A3R2 |
| SGK | LTF |
| SGK | KPNA2 |
| SGK | PA2G4 |
| SLC9A3R2 | SGKL |
| IGHA1 | CDK5RAP3 |
| IARS | FTS |
| IARS | BCAR3 |
| GSK3B | PTPN1 |
| SGTA | PRG1 |
| SGTA | EFEMP1 |
| SYT4 | TFAP2B |
| MAPK9 | ZBTB25 |
| FGFR1 | KPNB1 |
| BAX | SLC25A4 |
| ZAP70 | TUBA1 |
| DOK2 | SSSCA1 |
| DOK2 | CFLAR |
| DOK2 | ASF1B |
| NDP52 | SMARCD1 |
| NDP52 | GABARAPL2 |
| NDP52 | TCFL1 |
| NDP52 | PIAS4 |
| IGF1R | IGFBP3 |
| ITGB4 | ERBB2IP |
| ITGB4 | ITGB4BP |
| MAP4K1 | MAP3K7 |
| MAP4K1 | DBNL |
| MAP4K1 | MAP3K11 |
| SHFM1 | FLJ11305 |
| DOK5 | RET |
| SHMT2 | GNAS |
| SKP1A | CDCA3 |
| SKP1A | FBXO2 |
| TUBA1 | NCOA6 |
| UBE2D1 | RNF126 |
| DCC | PTK2B |
| RBBP8 | FXR2 |
| RBBP8 | LIMS1 |
| BOK | MCL1 |
| NCAM1 | CSPG3 |
| SMN1 | NOLA1 |
| SIRT2 | CDC14B |
| SIRT2 | ELL |
| PHB2 | CDC25B |
| SKB1 | SUPT5H |
| SKB1 | FBL |
| SKIL | CPNE4 |
| SKIL | CPNE1 |
| SKIL | CPNE2 |
| SMAD2 | DAB2 |
| SMAD3 | DAB2 |
| SMAD3 | CFDP1 |
| SMAD4 | CD59 |
| CDCA3 | CTDSP1 |
| BTRC | DLG7 |
| BTRC | UBE2D2 |
| CDK2 | CCNK |
| CDK2 | C20ORF81 |
| CDK2 | NPDC1 |
| E2F1 | NPDC1 |
| MYBL2 | UBB |
| SYK | UBB |
| VAV1 | EZH2 |
| BSG | MMP1 |
| ARHGEF11 | GNA12 |
| APPBP2 | CNTFR |
| SMARCA4 | ACTB |
| SMARCD2 | XRN1 |
| SURB7 | MED28 |
| USF2 | PTP4A2 |
| HIST2H3C | NASP |
| THRAP1 | MED28 |
| CRSP6 | MED28 |
| FANCA | CD79A |
| FANCA | GNB2 |
| FANCA | ATP5B |
| FHL2 | ITGA3 |
| AKT1 | CHN2 |
| AKT1 | MLLT7 |
| AKT1 | MTCP1 |
| HSPA8 | CITED1 |
| KPNA2 | ITK |
| LMO4 | LDB1 |
| MAP4K4 | SLC9A1 |
| MAP4K4 | RASA1 |
| MAP4K4 | ITGB1 |
| MSH6 | CASP4 |
| NMI | NECAP2 |
| POLR2K | TRG20 |
| POU2F1 | LIFR |
| BARD1 | UBE2D2 |
| RBL1 | TAF1 |
| RELA | KRIT1 |
| RELA | APBA2 |
| CSTF2 | DDX1 |
| CRSP3 | MED28 |
| CRSP3 | ELF3 |
| CANX | EBI3 |
| PTK2B | GNA13 |
| SLC3A2 | BRF2 |
| CA2 | SLC4A8 |
| ANK1 | AE2 |
| CFTR | SNAP23 |
| CAV3 | PFKM |
| TSC | RAI1 |
| TSC | TCTE1L |
| SLC9A2 | SPTA1 |
| RASA1 | AURKB |
| SLC9A3R1 | PAG1 |
| ADRB2 | GNA15 |
| PDGFRA | SNX6 |
| PDGFRA | ITGB3 |
| PDGFRB | EIF2AK2 |
| PDGFRB | ITGB3 |
| PTHR1 | PTH |
| RDX | CPNE2 |
| GRN | CCNT1 |
| GRN | HOXA1 |
| PLSCR1 | MGC3162 |
| PLSCR1 | C20ORF81 |
| PLSCR1 | C10ORF10 |
| PLSCR1 | HEY2 |
| PLSCR1 | HOXA9 |
| PHB | MAP3K10 |
| PHB | ANXA2 |
| STAT2 | IFNAR1 |
| STAT2 | IFNAR2 |
| CEBPB | HNRPK |
| YEATS4 | PFDN1 |
| SMN2 | SNRPE |
| SNRPB | DNCH1 |
| SNRPF | UQCRC1 |
| SNAP23 | NAPA |
| STX11 | SNAP25 |
| STX4A | VAMP4 |
| SYBL1 | STX7 |
| ABI3 | RUNX1T1 |
| FXR2 | AP2M1 |
| NAPA | STX5A |
| SNAP25 | TRIM9 |
| SNAP25 | STXBP1 |
| STXBP1 | APBA2 |
| SNAP29 | STX7 |
| AP2A1 | ENTH |
| 4-Sep | 8-Sep |
| DNCL1 | NEK6 |
| DNCL1 | MTA1 |
| DNCL1 | DNMT3A |
| CRP | FCGR1A |
| CRP | FCGR2A |
| CRP | FCGR2B |
| CTDP1 | MEP50 |
| PDLIM7 | PSMF1 |
| PDLIM7 | C1ORF65 |
| PDLIM7 | ZNF165 |
| FBL | HRMT1L2 |
| DTNA | DTNBP1 |
| MAPK12 | DUSP1 |
| ABCA1 | APOA1 |
| SNX6 | LEPR |
| ACVR2B | SYNJ2BP |
| SSR4 | HECW1 |
| TRIP6 | FLJ22746 |
| TRIP6 | HOXA9 |
| TRIP6 | AQP1 |
| TRIP6 | NEDD9 |
| SORT1 | NGFB |
| NCK2 | FLJ12529 |
| NCK2 | KIAA1217 |
| PSMC5 | TNNT1 |
| SPARC | VEGF |
| THBS1 | CD36 |
| COL3A1 | DDR2 |
| MMP14 | C1QBP |
| GDI1 | RAB9A |
| FANCC | TZFP |
| ACTB | VSNL1 |
| ACTB | PLD2 |
| EPB42 | ECG2 |
| SYN1 | CAPON |
| CPNE4 | WTAP |
| CPNE4 | PITPNM2 |
| CPNE1 | WTAP |
| CPNE1 | PITPNM2 |
| KIFAP3 | CSPG6 |
| HRMT1L2 | SSSCA1 |
| HRMT1L2 | IFNAR1 |
| HRMT1L2 | CIRBP |
| HRMT1L2 | KHDRBS2 |
| HRMT1L2 | KHDRBS1 |
| NCL | CD3E |
| NCL | MDK |
| SSSCA1 | CCNH |
| SSSCA1 | CCT3 |
| MGC2650 | EFS |
| SST | SSTR5 |
| SST | SSTR2 |
| SSTR5 | CORT |
| CORT | SSTR2 |
| HSPCA | RUNX1T1 |
| L3MBTL2 | FLJ11730 |
| IL27RA | EBI3 |
| TSHR | JAK1 |
| TSHR | GNA13 |
| TSHR | HSPA5 |
| TSHR | JAK2 |
| STAT4 | IL12RB2 |
| TEBP | COPG |
| PRNP | PLG |
| STK6 | CDC20 |
| STK6 | NIN |
| CDC20 | HSPC128 |
| CDC20 | MAD2L2 |
| NAGK | DACH1 |
| MBP | MMP7 |
| MBP | MAG |
| MGC2749 | ING5 |
| MGC2749 | TPM3 |
| 5-Sep | 8-Sep |
| RAB11A | RAB3IL1 |
| MAP3K4 | MAPK13 |
| BLNK | CD79A |
| CD3E | TOP2B |
| CD3E | CD3EAP |
| EPOR | MAPK3 |
| FCGR1A | HRG |
| FCGR1A | APCS |
| FGR | NCOA6 |
| JAK1 | PLAUR |
| TDRD7 | GABPB2 |
| AKAP9 | PPP2R1B |
| EXOC8 | SEC8L1 |
| TU3A | PPP2R2A |
| UBTF | SMURF1 |
| UBTF | SERPINH1 |
| LMO2 | MLLT4 |
| TRAF3 | TRIM37 |
| IKBKE | HSPCAL3 |
| IKBKE | KTN1 |
| IKBKE | PPP2R1A |
| TRIM37 | MGC33889 |
| TRIM37 | PBK |
| TUBB2 | MX1 |
| TBL1X | GPS2 |
| BRF2 | HSHIN1 |
| BRF2 | SMAP1 |
| BRF2 | HSPC121 |
| CREG1 | IGF2R |
| CREM | CSEN |
| NFYB | CNTN2 |
| TCAP | GDF8 |
| TCEAL1 | SSR3 |
| SSR3 | C20ORF24 |
| PPP2R2A | ATR |
| PPP2R2A | PPP2R1B |
| TCEB1 | LRRC41 |
| TCEB1 | SOCS3 |
| TCEB1 | EXT2 |
| TCEB1 | GC20 |
| TCEB1 | SOCS1 |
| TCEB2 | LRRC41 |
| TCEB2 | SOCS1 |
| TCF1 | PCBD1 |
| TCF1 | ALB |
| RAC3 | NRBP |
| CALM2 | CALD1 |
| CALM2 | KIAA1683 |
| MLX | MNT |
| VDAC1 | DCD |
| VDAC1 | GSN |
| VDAC1 | HSPA9B |
| PVR | AP1M2 |
| MAX | SPAG9 |
| TEAD2 | YAP1 |
| YAP1 | TEAD4 |
| YAP1 | TP53BP2 |
| YAP1 | WBP1 |
| YAP1 | RUNX2 |
| YAP1 | ERBB4 |
| XRCC5 | CD40 |
| TF | FNBP1 |
| TFRC | CD3Z |
| TFAP2A | LDLRAP1 |
| EIF3S10 | EIF3S9 |
| EIF3S10 | EIF3S3 |
| TG | ASGR1 |
| TG | TRA1 |
| ASGR1 | DHFR |
| SMURF1 | MAP3K7 |
| SMURF1 | ETV6 |
| SMURF1 | APBB2 |
| SMURF1 | RAPSN |
| DAB2 | PIN1 |
| EFNA1 | EPHA5 |
| OCLN | YES1 |
| CDK6 | PPM1B |
| RAB33B | RABEP1 |
| DAPK2 | CAMK2A |
| UBD | MAD2L1 |
| UBD | NYREN18 |
| GIT2 | PAK2 |
| GIT2 | ADRBK1 |
| CSNK1D | MCC |
| CSNK1D | GJA1 |
| NEK6 | PSMD2 |
| NEK6 | EFCBP2 |
| TTC1 | GNAS |
| DUSP10 | MAPK11 |
| RNF138 | C6ORF165 |
| ARF4L | ARL6IP |
| GNA13 | PPP5C |
| CHUK | TRAF3IP2 |
| ITK | HNRPK |
| TGIF | CTBP2 |
| CALML5 | YWHAQ |
| F2 | SERPINB8 |
| CD36 | MATK |
| FGA | BAT2 |
| LRP5 | AXIN1 |
| LRP5 | DKK1 |
| TIAM1 | CAMK2G |
| MAPK8IP2 | MAPK13 |
| FLJ36766 | VSNL1 |
| FLJ36766 | MAD1L1 |
| TREX1 | IFT20 |
| TIMM13 | PREI3 |
| PREI3 | HSPC128 |
| TIMP2 | PSMA7 |
| TIMP3 | ADAM17 |
| ITGB1 | FLT4 |
| TRA1 | HSPA9B |
| CD14 | DAF |
| CD81 | IFITM1 |
| ITGA3 | LGALS8 |
| ITGA6 | RPSA |
| HSHIN1 | APG12L |
| TNFRSF10A | DAP3 |
| TNFRSF10A | BCL10 |
| CD40 | NCOA6 |
| TRAF3IP2 | MDFI |
| POLR2H | IXL |
| POLR2H | CRSP7 |
| POLR2H | TRG20 |
| PDCD6 | C14ORF32 |
| PDCD6 | CPNE2 |
| IL1RN | IL1R1 |
| NUMA1 | EPB41L1 |
| NUMA1 | GPSM2 |
| TNNI3 | PKD2 |
| TNNI3 | PAPPA |
| TNNT1 | PRKG1 |
| TNNT1 | PPFIA1 |
| TOB1 | CNOT7 |
| MARCKS | CTSB |
| MAPK1 | SCAM-1 |
| PIN1 | PTPN1 |
| TOP3B | FBLN5 |
| ZNF408 | MIF |
| UBE2A | RFPL4 |
| CCNH | AP2A2 |
| TSG101 | KRT15 |
| ING5 | MCM6 |
| ELL | EAP30 |
| CR2 | CR1 |
| PRKRA | PRPSAP1 |
| BAK1 | DFFA |
| CSNK1E | MCC |
| TFDP1 | NPDC1 |
| GORASP2 | CBLB |
| SOC | C6ORF165 |
| SOC | RND1 |
| ZNF297B | ARRB1 |
| IL15RA | IL2RB |
| PSMF1 | CTBP2 |
| IRAK2 | IL1R1 |
| ACP1 | KDR |
| MYO1C | WDR39 |
| MMS19L | PTP4A3 |
| ATR | PIK3CA |
| ATR | ARHGEF1 |
| ATR | AP2A2 |
| ATR | FLT1 |
| MOBK1B | NUP98 |
| ND1 | DHDDS |
| GNAS | ADCY5 |
| DNAJC7 | HUS1 |
| DNAJC7 | RAD9A |
| TTR | ATF4 |
| APOA1 | PDE1A |
| C1QA | CR1 |
| MARK4 | MAP2 |
| MARK4 | MYH9 |
| BAT2 | HNRPM |
| NXF1 | ELAVL4 |
| UBE2D2 | PJA1 |
| UBE2G1 | SMURF2 |
| AUP1 | ITGA2B |
| AUP1 | ITGAV |
| ZNFN1A3 | ZNFN1A5 |
| HIPK2 | TP73L |
| RAD54B | CDC23 |
| ZNF451 | RAP1GDS1 |
| UBE2M | APPBP1 |
| UBE2M | PRKAR1A |
| KLHDC5 | SERTAD1 |
| PSEN1 | GFAP |
| TOMM20 | DHFR |
| GTF2I | PTP4A3 |
| PTP4A2 | RABGGTB |
| FLJ11588 | CTNNBIP1 |
| BCAP31 | CASP1 |
| VASP | DMRTB1 |
| DMRTB1 | HRMT1L1 |
| NOL3 | PTPNS1 |
| PRKCQ | TXNL2 |
| RHOG | PLD1 |
| RHOG | KTN1 |
| RHOG | ARHGDIG |
| ITGB7 | EED |
| DCD | RAD51L3 |
| DCD | CD2BP2 |
| DCD | EIF2B1 |
| VEGF | FLT1 |
| KDR | ANXA5 |
| KDR | COL18A1 |
| RAB1B | GTP |
| CNTF | CNTFR |
| CNTF | LIFR |
| CSTB | CTSD |
| IMPDH2 | APG5L |
| MCC | DFFA |
| MCC | PPP2R1A |
| PPIB | CGI-119 |
| L1CAM | CSPG3 |
| VIPR1 | WASL |
| CLU | LRP2 |
| LRPAP1 | SORL1 |
| VSNL1 | CHRNA4 |
| IGFBP5 | KPNB1 |
| KNG1 | FTL |
| KNG1 | KRT1 |
| GP1BA | YWHAZ |
| HSPA5 | STMN1 |
| F8 | PHYH |
| ACTR3 | GC20 |
| KIAA1967 | WDR39 |
| ARMC6 | STK36 |
| PHYH | FKBP4 |
| PHYH | MAGEA11 |
| WNT5A | FZD5 |
| CETN2 | RAD23B |
| KIF17 | APBA1 |
| RAN | DDEF2 |
| HDLBP | HSD11B1 |
| CD2AP | CD2 |
| MAP3K10 | DNM1L |
| RGS3 | EFNB1 |
| YWHAH | RIMS2 |
| YWHAQ | PAK6 |
| DDX6 | PAF53 |
| PIK3CA | NRAS |
| YWHAZ | FOXO1A |
| YWHAZ | FOXO3A |
| CSF2RB | MAD2L1 |
| VIM | MYST2 |
| AGTR1 | AGT |
| DDX17 | SCAM-1 |
| FLJ20626 | ZNF24 |
| FLJ20626 | ZNF496 |
| NME7 | FKBP6 |
| SCAM-1 | CBLB |
| SCAM-1 | EFS |
| ZNF9 | RBMX |
| HNRPAB | AES |
| RBMX | HNRPK |
| ZNFN1A2 | ZNFN1A5 |
| ZNFN1A4 | ZNFN1A5 |
| NCOR2 | SKIIP |
| C2 | PSMA4 |
| STX18 | RINT-1 |
| PLD2 | ALDOA |
| TPM3 | CNN1 |
| EMD | C10ORF30 |
| ALS2CR3 | KCNJ2 |
| BPY2 | BPY2IP1 |
| CLK4 | HSPB2 |
| AP1M2 | LDLR |
| SURF4 | C20ORF24 |
| CALCOCO1 | PSCD3 |
| NCOA6 | CXADR |
| DISC1 | EIF3S3 |
| HSPD1 | DHFR |
| FLJ12529 | HRMT1L1 |
| WWP1 | WBP1 |
| WWP1 | ATN1 |
| KLHL12 | MED28 |
| KLHL12 | KNTC2 |
| ARMC7 | KCTD13 |
| HNRPM | CEACAM5 |
| CDK2AP1 | IGHA2 |
| IGHA2 | ARK5 |
| POLA | SERPINA3 |
| LPXN | PHF21A |
| LPXN | ITGA4 |
| WDR39 | CCDC7 |
| SYNE1 | MUSK |
| ANXA5 | EED |
| CALML3 | MYO10 |
| SPAG9 | EIF4A2 |
| CD1D | CD74 |
| PRF1 | GZMB |
| MTMR9 | EMILIN1 |
| CCND3 | AREG |
| MBTPS1 | CREBL1 |
| EFCBP2 | RUNX1T1 |
| GTP | RAPGEF6 |
| GTP | MX1 |
| CART1 | ALX4 |
| IL16 | PPP1R12A |
| PPP1CB | TMEM33 |
| CASP1 | CARD4 |
| CARD12 | PYCARD |
| CASP4 | USP9X |
| CSEN | PRV1 |
| KIAA0776 | CDK5RAP3 |
| EIF2S3 | METAP2 |
| APAF1 | MMRP19 |
| CAT | ABL2 |
| SHOC2 | NRAS |
| SERPINH1 | CD9 |
| IL1F5 | SSBP2 |
| IL1F5 | MCM6 |
| ARHGEF15 | EPHA4 |
| PRAM-1 | MXD1 |
| DKFZP564J157 | PPIA |
| RASD1 | CAPON |
| KDELR1 | WBP5 |
| KDELR1 | BZW1 |
| FLJ10719 | ILK |
| HMG20A | DTNB |
| WNT3A | PORCN |
| GRIPAP1 | GRIP2 |
| CCR2 | IRF2 |
| ITGB5 | ZBTB17 |
| ITGB5 | LTBP1 |
| PGRMC1 | EFHD1 |
| HIRA | H3F3B |
| ACTN1 | FBP1 |
| PDLIM1 | RNF12 |
| ADORA2A | ADA |
| MAGEA11 | ARHGAP29 |
| AP2M1 | CTLA4 |
| AP2M1 | RTDR1 |
| NID | PTPRF |
| CD2BP2 | USP39 |
| CD2BP2 | CD2 |
| CD2 | MXD1 |
| SKIIP | LRP2BP |
| RNF12 | ISL1 |
| GNG11 | AMOTL2 |
| RAB14 | RIPK5 |
| CD74 | MIF |
| GSTK1 | XPO5 |
| IGF2R | PLAU |
| KNTC2 | AMOTL2 |
| KNTC2 | PLDN |
| AMOTL2 | MAD1L1 |
| MAD2L1 | RIPK5 |
| PLDN | BLOC1S1 |
| GOT2 | MPG |
| CPVL | PLRG1 |
| MGC10540 | MMRP19 |
| CS | CRYAA |
| ARFIP2 | ARL1 |
| EEF1D | KTN1 |
| FANCG | KRT19 |
| COPG | DRD1 |
| MYO1B | EIF4A2 |
| ITGAE | EED |
| DLC2 | BMF |
| EED | ITGA4 |
| DUSP16 | MAPK11 |
| TRPM7 | PLCB2 |
| KHDRBS2 | HNRPK |
| GHRL | GHSR |
| RND2 | MEOX2 |
| CEBPG | GRINL1A |
| LRRK1 | LYZ |
| SFI1 | KRT19 |
| DXYS155E | EIF4A2 |
| TNK2 | MCF2 |
| MED28 | CRSP2 |
| MED28 | CRSP9 |
| FGFBP1 | FIBP |
| CNKSR1 | RHPN1 |
| PCQAP | IXL |
| PCQAP | CRSP7 |
| PCQAP | MED9 |
| PCQAP | TRG20 |
| PCQAP | MED25 |
| PCQAP | MED19 |
| PCQAP | FHL3 |
| VDAC2 | TDE1 |
| LAMA5 | MEP1A |
| FGF7 | DUSP3 |
| COL8A1 | ITGA1 |
| COL8A1 | ITGA2 |
| IXL | CRSP9 |
| IXL | POLR2L |
| CRSP7 | CRSP9 |
| CRSP7 | POLR2L |
| CRSP9 | MED9 |
| CRSP9 | TRG20 |
| CRSP9 | MED19 |
| TRG20 | POLR2L |
| POLR2L | NOV |
| HTATIP | TCF8 |
| DDX5 | STK24 |
| FCGR2B | APCS |
| UBADC1 | IQGAP2 |
| UBADC1 | MYH2 |
| UBADC1 | EIF4EBP1 |
| MDFI | EBI3 |
| XK | KEL |
| ECG2 | MT2A |
| GMIP | GEM |
| FLJ25393 | IFIT3 |
| CTBP2 | TCF8 |
| DDX19L | AD023 |
| ZCCHC10 | STAC3 |
| EIF2B1 | PDIA4 |
| TXNL5 | OXT |
| FREQ | DRD5 |
| ACP6 | DHCR7 |
| NFASC | DCX |
| IL1R1 | IL1B |
| GC20 | EIF2S1 |
| GC20 | RAB7 |
| GC20 | EIF3S3 |
| EIF3S3 | EIF4A2 |
| EPHA5 | EFNA3 |
| EIF4G2 | MKNK1 |
| ATIC | NDUFAF1 |
| MYOC | MYL2 |
| FGFR4 | FGF8 |
| FPR1 | ANXA1 |
| CP | ATP7A |
| GDI2 | RAB9A |
| GH1 | LTA4H |
| GLE1L | NUP155 |
| A2M | PAEP |
| MAGI3 | PTPRB |
| RGS12 | GRM5 |
| HBB | HPR |
| HOXB6 | KRT15 |
| AHSA1 | PHLDA3 |
| IL10 | IL10RA |
| MYO1D | PELO |
| PSCD1 | TRIM23 |
| PSCD1 | ARFRP1 |
| KRT17 | KRT6A |
| RPL28 | MAGEB2 |
| STK24 | ZBTB24 |
| MLF2 | BAG2 |
| PRKG1 | NPR1 |
| NEF3 | NEFH |
| PLA2G1B | PLAA |
