# Supplementary material for: Geometric De-noising of Protein-Protein Interaction Networks
Source: PLoS Comput Biol. 2009 Aug 7;5(8):e1000454. doi: 10.1371/journal.pcbi.1000454 (PMC2711306; doi:10.1371/journal.pcbi.1000454)
Supplement: Table S7 — Experimental techniques from BIOGRID capable of detecting physical interactions between proteins. (0.04 MB DOC) [file pcbi.1000454.s008.doc]

**Table S7: Experimental techniques from BIOGRID capable of detecting physical interactions between proteins.**

| Affinity Capture-Luminescence |
| --- |
| Affinity Capture-MS |
| Affinity Capture-RNA |
| Affinity Capture-Western |
| Biochemical Activity |
| Co-crystal Structure |
| Co-fractionation |
| Co-localization |
| Co-purification |
| Far Western |
| FRET |
| Invitro |
| Invivo |
| PCA |
| Protein-peptide |
| Protein-RNA |
| Reconstituted Complex |
| Two-hybrid |
