# Supplementary figures and images for: Geometric De-noising of Protein-Protein Interaction Networks
Source: PLoS Comput Biol. 2009 Aug 7;5(8):e1000454. doi: 10.1371/journal.pcbi.1000454 (PMC2711306; doi:10.1371/journal.pcbi.1000454)

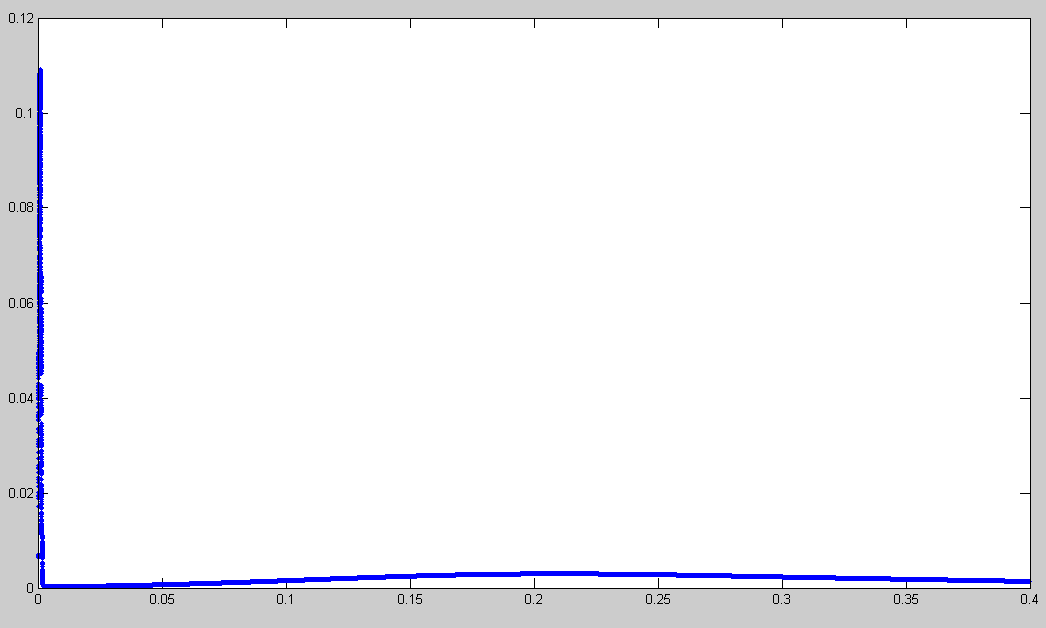

Supplement: Figure S1 — Probabilistic density p(edge|dist). x axis corresponds to distances between pairs of nodes, y value of the density. Note, that in this plot normalization constant from formula (7) in the main paper is not taken into account. (0.02 MB TIF) [file pcbi.1000454.s009.tif]

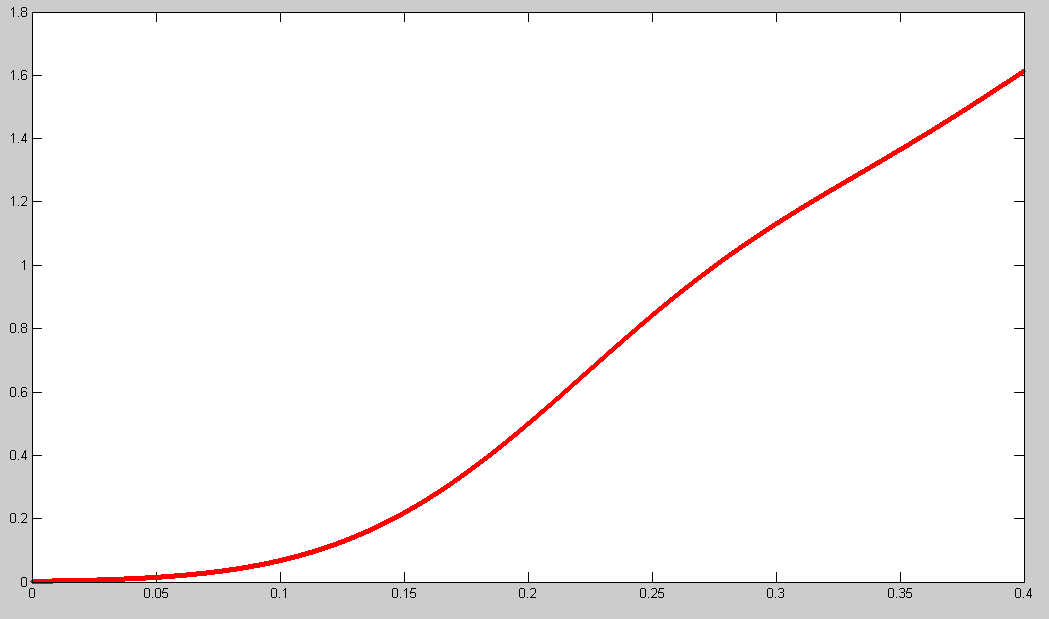

Supplement: Figure S2 — Probabilistic density p(nonedge|dist). x axis corresponds to distances between pairs of nodes, y value of the density. Note, that in this plot normalization constant from formula (8) in the main paper is not taken into account. (0.02 MB TIF) [file pcbi.1000454.s010.tif]

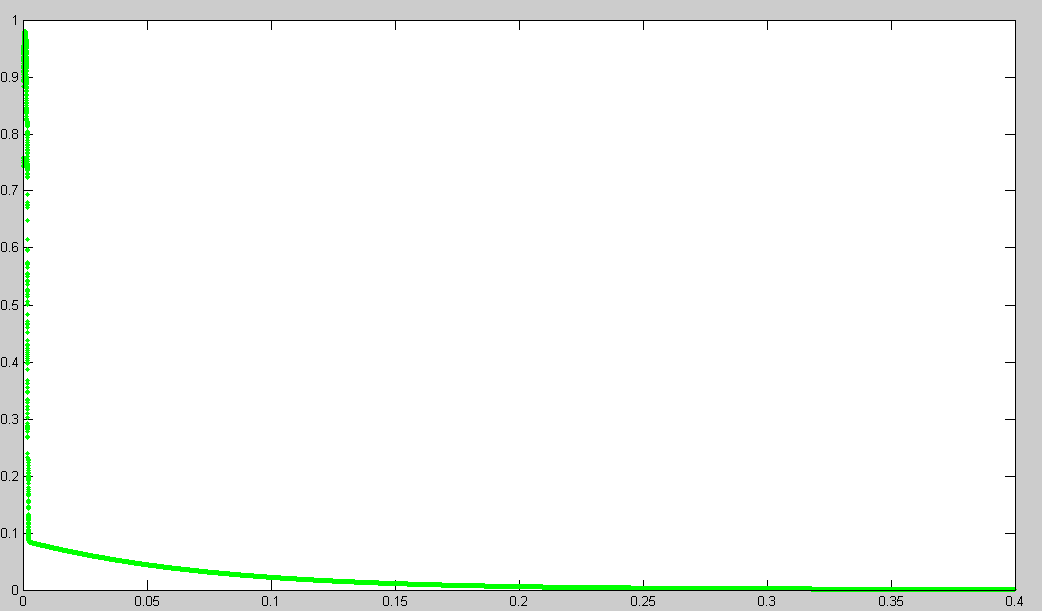

Supplement: Figure S3 — Confidence scores for “HumanBG network”. x axis corresponds to distances between pairs od nodes, y axis corresponds to the assigned confidence scores. (0.02 MB TIF) [file pcbi.1000454.s011.tif]
